# Worldwide population differentiation at disease-associated SNPs

**DOI:** 10.1186/1755-8794-1-22

**Published:** 2008-06-04

**Authors:** Sean Myles, Dan Davison, Jeffrey Barrett, Mark Stoneking, Nic Timpson

**Affiliations:** 1 Department of Evolutionary Genetics, Max Planck Institute for Evolutionary Anthropology, Deutscher Platz 6, 04103 Leipzig, Germany; 2 Department of Statistics, Oxford University, 1 South Parks Road, Oxford, OX1 3TG, UK; 3 Wellcome Trust Centre for Human Genetics, Roosevelt Drive, Oxford, OX3 7BN, UK; 4 MRC CAiTE Centre, Department of Social Medicine, University of Bristol, Canynge Hall, Whiteladies Road, Bristol, BS8 2PR, UK; 5 Institute for Genomic Diversity, Cornell University, 175 Biotechnology Building, Ithaca, NY 14853-2703, USA

## Abstract

**Background:**

Recent genome-wide association (GWA) studies have provided compelling evidence of association between genetic variants and common complex diseases. These studies have made use of cases and controls almost exclusively from populations of European ancestry and little is known about the frequency of risk alleles in other populations. The present study addresses the transferability of disease associations across human populations by examining levels of population differentiation at disease-associated single nucleotide polymorphisms (SNPs).

**Methods:**

We genotyped ~1000 individuals from 53 populations worldwide at 25 SNPs which show robust association with 6 complex human diseases (Crohn's disease, type 1 diabetes, type 2 diabetes, rheumatoid arthritis, coronary artery disease and obesity). Allele frequency differences between populations for these SNPs were measured using Fst. The Fst values for the disease-associated SNPs were compared to Fst values from 2750 random SNPs typed in the same set of individuals.

**Results:**

On average, disease SNPs are not significantly more differentiated between populations than random SNPs in the genome. Risk allele frequencies, however, do show substantial variation across human populations and may contribute to differences in disease prevalence between populations. We demonstrate that, in some cases, risk allele frequency differences are unusually high compared to random SNPs and may be due to the action of local (i.e. geographically-restricted) positive natural selection. Moreover, some risk alleles were absent or fixed in a population, which implies that risk alleles identified in one population do not necessarily account for disease prevalence in all human populations.

**Conclusion:**

Although differences in risk allele frequencies between human populations are not unusually large and are thus likely not due to positive local selection, there is substantial variation in risk allele frequencies between populations which may account for differences in disease prevalence between human populations.

## Background

A broadly accepted model for the genetic architecture of complex disease is the common disease – common variant (CDCV) hypothesis. This hypothesis proposes that risk alleles for common complex diseases should be common (i.e. ≥ 5%) and thus are likely old and found in multiple human populations, rather than being population specific [1-4]. From analyses of genome-wide polymorphism data from populations of African, Asian and European ancestry, it has been shown that common alleles in one population are frequently both shared and common among human populations [5-7]. However, a recent comprehensive study of 3,873 genes from African, Asian, Latino/Hispanic, and European Americans found that common alleles in one population were frequently not common in another population [8]. Similarly, from a meta-analysis of disease-association studies, Ioannidis et al. (2004) argued that the frequencies of disease-associated alleles show "large heterogeneity between races" [9]. These observations suggest that the frequency of a risk allele discovered in one population is not always a strong predictor of the frequency of that risk allele in other populations. This raises the question of whether risk alleles discovered in one population account for disease prevalence across all human populations. Thus, it remains unknown how well the CDCV model accounts for disease prevalence across populations on a worldwide scale.

In addition to evaluating the extent to which disease-associated alleles differ in frequency between populations, it is of great interest to determine what evolutionary forces are responsible for the observed degree of population differentiation at disease-associated SNPs. Because disease is so tightly linked to survival and reproductive success, it follows that disease has likely been a strong selective force in human evolution. Moreover, alleles that cause disease in contemporary environments may have been positively selected in ancestral environments. For example, the thrifty gene hypothesis posits that populations whose ancestral environments were characterized by periods of feast and famine may have experienced selection for a "thrifty genotype" that promotes efficient fat and carbohydrate storage [10]. Though formerly advantageous, thrifty genotypes may be causing obesity and type 2 diabetes in contemporary environments where food is often abundantly available. Previous studies have suggested that genes associated with complex diseases such as cardiovascular disease [11-14] and type 2 diabetes [15-17] have been targets of positive natural selection. If disease genes have often been targeted by selection, then identifying loci that have experienced selection may aid in disease-related research [18].

Local (i.e. geographically-restricted) positive selection results in large allele frequency differences between populations [e.g. [19,20]]. The Fst statistic captures the difference in allele frequency between populations at any given SNP and ranges from 0 (no differentiation) to 1 (fixed difference between populations). Thus, when compared to a set of random SNPs in the genome, positively selected alleles tend to accumulate in the top tail of the Fst distribution [21-23]. It has previously been shown that local positive selection has had no widespread effect on disease allele frequency differences between populations: on average, disease-associated SNPs showed allele frequency differences between populations similar to those observed for random SNPs [24]. Individually, however, several disease-associated alleles appear to have been driven to high frequency by positive selection in certain human populations and thus may be responsible for large differences in disease prevalence between populations [15,25].

The conclusions drawn from previous studies that have evaluated levels of population differentiation at disease-associated SNPs are limited for two reasons. First, these studies relied on many disease-gene associations that have not been successfully replicated and thus likely do not represent true associations. Second, previous studies made use of disease allele frequencies from a small number of populations (i.e. ≤ 4). To address the strength of the CDCV model on a worldwide scale and to evaluate the effects of local positive selection on worldwide risk allele frequencies, we present allele frequencies and levels of population differentiation across 53 populations for 25 SNPs which show replicated association with the following common complex human diseases: Crohn's disease, type 1 diabetes, type 2 diabetes, rheumatoid arthritis, coronary artery disease and obesity [17,26-42]. These newly identified genetic variants came from recent genome-wide association (GWA) study data, which have revolutionized approaches for identifying disease loci [43].

## Methods

The 25 SNPs from Table 1 were genotyped in the HGDP-CEPH Panel [44]. Atypical and related individuals were removed [45], which resulted in 952 individuals from 53 populations. SNPs were genotyped by KBioscience using the KASPar chemistry, a competitive allele specific PCR SNP genotyping system [46].

**Table 1:** Worldwide risk allele frequencies and global Fst for 25 disease-associated SNPs typed in the CEPH-HGDP panel.

									Risk allele frequency							
SNP^1^	Disease^2^	Replication	Chr	Position^3^	Gene^4^	Global Fst	*P*	*P*_*cor*_	Global	Africa	Middle East	Europe	Central South Asia	East Asia	America	Oceania

rs10077785	CD	[30]	5	131829057		0.062	0.642	0.511	0.82	0.975	0.809	0.812	0.716	0.898	0.688	0.75
rs10210302	CD	[30]	2	233940839	ATG16L1	0.117	0.315	0.323	0.393	0.268	0.459	0.539	0.541	0.31	0.066	0.018
rs10761659	CD	[30]	10	64115570		0.251	0.036	0.046	0.542	0.015	0.427	0.507	0.631	0.759	0.811	0.269
rs10811661	T2D	[27]	9	22124094	CDKN2A/2B	0.126	0.278	0.224	0.782	0.97	0.805	0.833	0.876	0.584	0.836	0.518
rs10883365	CD	[29]	10	101277754		0.04	0.8	0.65	0.459	0.48	0.541	0.497	0.43	0.449	0.161	0.643
rs10946398	T2D	[27, 31]	6	20769013	CDKAL1	0.028	0.901	0.697	0.328	0.47	0.338	0.286	0.243	0.382	0.242	0.321
rs1111875	T2D	[27, 28]	1	218111919		0.179	0.131	0.183	0.525	0.828	0.664	0.588	0.487	0.232	0.685	0.554
rs11171739	T1D	[32]	12	54756892		0.221	0.063	0.049	0.367	0.884	0.343	0.438	0.318	0.219	0.056	0.481
rs11805303	CD	[33]	1	67387537	IL23	0.085	0.483	0.492	0.421	0.27	0.456	0.303	0.513	0.547	0.121	0.446
rs12708716	T1D	[32]	16	11087374	KIAA0350	0.071	0.57	0.398	0.648	0.411	0.592	0.611	0.645	0.773	0.849	0.571
rs13266634	T2D	[27, 28]	8	114748339	SLC30A8	0.07	0.575	0.365	0.74	0.941	0.803	0.721	0.756	0.593	0.703	0.911
rs1333049	CAD	[34, 35, 36]	9	22115503		0.079	0.516	0.464	0.483	0.157	0.54	0.569	0.536	0.52	0.5	0.161
rs17234657	CD	[37]	5	40437266		0.112	0.334	0.192	0.07	0.243	0.099	0.126	0.021	0.002	0.008	0
rs17696736	T1D	[32]	12	110949538	C12orf30	0.237	0.049	0.113	0.165	0	0.37	0.413	0.13	0.011	0.04	0
rs1801282	T2D	[27, 38, 39]	3	12368125	PPARG	0.021	0.943	0.581	0.923	1	0.938	0.91	0.877	0.923	0.897	1
rs2542151	T1D/CD	[29]	18	12769947		0.008	0.991	0.77	0.153	0.183	0.127	0.144	0.179	0.154	0.172	0.018
rs4402960	T2D	[27]	3	186994389	IGF2BP2	0.077	0.53	0.612	0.371	0.693	0.302	0.354	0.378	0.306	0.218	0.536
rs5215	T2D	[27, 38, 39]	11	17365206	KCNJ11	0.057	0.671	0.697	0.319	0.056	0.268	0.418	0.34	0.377	0.312	0.393
rs564398	T2D	[27]	9	22019547	CDKN2A/2B	0.113	0.332	0.246	0.818	1	0.848	0.706	0.753	0.862	0.937	0.34
rs6679677	T1D/RA	[40, 41]	1	114015850	RSBN1	0.019	0.95	0.875	0.016	0	0.019	0.055	0.013	0.004	0	0
rs6887695	CD	[29]	5	158755223		0.028	0.898	0.741	0.362	0.381	0.383	0.281	0.299	0.409	0.371	0.643
rs7901695	T2D	[27, 28, 31]	10	114744078	TCF7L2	0.213	0.073	0.08	0.281	0.629	0.438	0.325	0.321	0.044	0.087	0.054
rs9858542	CD	[29]	3	49676987	BSN	0.094	0.432	0.318	0.222	0.23	0.301	0.317	0.331	0.077	0.016	0.143
rs9939609	T2D/OB	[27, 42]	16	52378028	FTO	0.101	0.391	0.446	0.315	0.471	0.41	0.426	0.348	0.157	0.048	0.25

^1 ^All SNPs were initially obtained from the WTCCC [26], except rs13266634 which was not well tagged by the Affymetrix GeneChip Human Mapping 500 K platform but was reported elsewhere as a T2D candidate [27, 28].

^2 ^CD = Crohn's disease; T2D = type 2 diabetes; T1D = type 1 diabetes; CAD = coronary artery disease; RA = rheumatoid arthritis; OB = obesity.

^3 ^Positions refer to NCBI Build 35 coordinates.

^4 ^Blank cells indicate that the SNP does not fall within or near a known coding gene.

All of the genotype calls were confirmed by visual inspection. After Bonferroni correction for 25 comparisons, there remained 4 SNPs for which a population was out of Hardy-Weinberg equilibrium at p < 0.002. The genotype calls in these cases were re-confirmed by visual inspection of the cluster plots and no data were removed. The amount of missing data per SNP ranged from 2.0% – 5.4% with a mean of 3.3%. These data are accessible from the CEPH database [47] or by request to the corresponding author.

Global Fst [48], the degree of differentiation among the 7 geographic regions represented in the CEPH-HGDP panel, was calculated for each of the 25 SNPs. Results were largely the same when global Fst was calculated among all 53 populations. We obtained an empirical Fst distribution from 2750 autosomal markers (2540 SNPs [49] and 210 indels [50]) previously typed in 927 individuals from the CEPH-HGDP panel. Global Fst values for the disease-associated SNPs were calculated from the same set of 927 individuals to allow for an unbiased comparison to the empirical distribution. For each disease-associated SNP, a *P *value was calculated as the proportion of Fst values from the empirical distribution that were ≥ the observed Fst value. We found that global Fst is weakly but significantly correlated with global minor allele frequency (R^2 ^= 0.0152, *P *= 5.04 × 10^-23^, see Additional file 1) and that the Fst distribution often differs significantly between minor allele frequency bins (Additional file 2). We therefore provide corrected *P *values (*P*_*cor*_) for each Fst value by comparing only to SNPs from the empirical distribution that fall into the same minor allele frequency bin.

## Results

**Figure 1:**
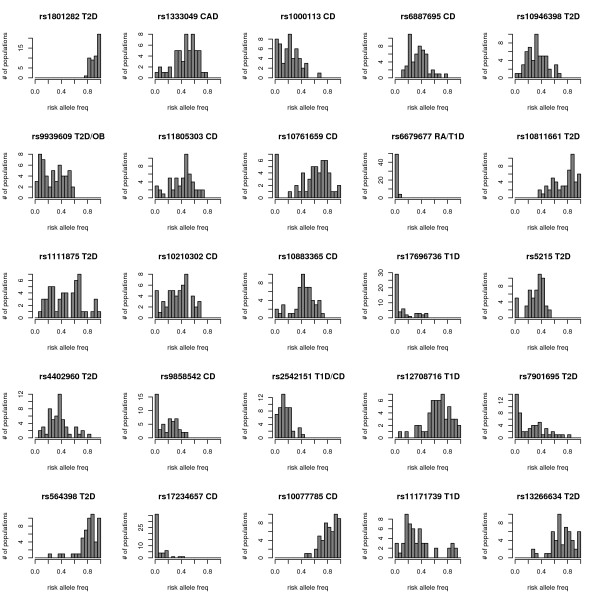
**Risk allele frequency across populations for 25 disease-associated SNPs.** The title of each histogram includes the dbSNP ID and the disease with which each SNP is associated. Abbreviations for disease names can be found in Table 1. Note that the Y axes have different scales across histograms.

**Figure 2:**
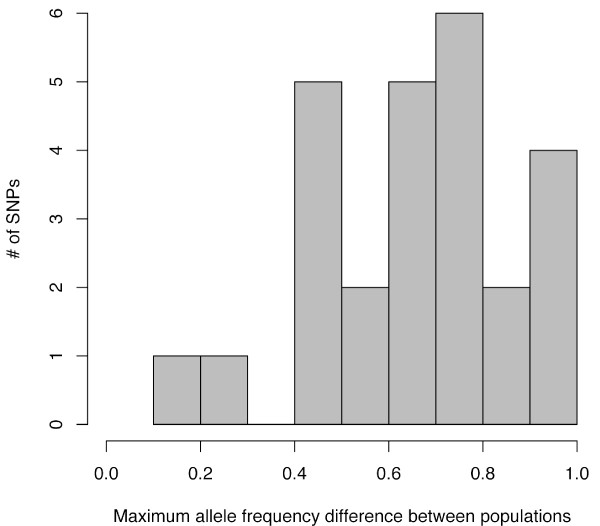
The maximum difference in risk allele frequency between any 2 of the 53 populations in the CEPH-HGDP panel across the 25 disease-associated SNPs.

We genotyped the HGDP-CEPH Human GenomeDiversity Cell Line Panel [44] for 25 disease-associated SNPs recently identified from GWA studies [26-28]. The global and regional allele frequencies for each disease-associated SNP are summarized in Table 1. To visualize worldwide risk allele frequencies, Figure 1 shows the allele frequency distribution across populations for each disease-associated SNP. A summary of the maximum allele frequency difference between any 2 of the 53 populations for each disease-associated SNP is presented in Figure 2.

**Figure 3:**
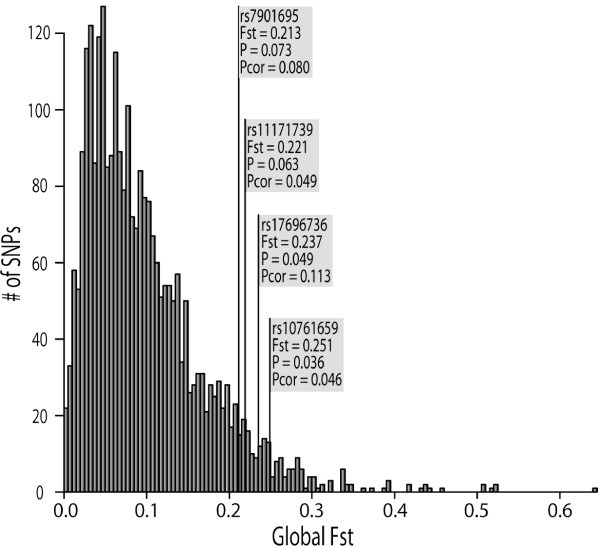
**Empirical global Fst distribution of 2750 markers typed in 927 individuals from the CEPH-HGDP panel.** Disease-associated SNPs with global Fst values within the top 10% of the empirical distribution are indicated.

For each disease-associated SNP, global Fst, a measure of allele frequency difference, was calculated among the 7 geographical regions represented in the HGDP-CEPH panel. It has been shown from empirical data and from simulations with varying parameters that alleles that have been targets of local positive selection tend to accumulate in the top tail of the Fst distribution [19-23]. Uncorrected *P *values (*P*) and *P *values corrected for allele frequency (*P*_*cor*_) were generated by comparing each observed global Fst value to an empirical global Fst distribution from 2750 markers typed in the same samples (see Materials and Methods for details). The global Fst value and the corresponding *P *value for each of the 25 disease-associated SNPs are summarized in Table 1. The empirical global Fst distribution is shown in Figure 3 along with the 4 most highly differentiated disease-associated SNPs (i.e. SNPs with uncorrected *P *values < 0.1).

To determine whether the mean global Fst of 0.100 for the 25 disease-associated SNPs is unusually high, this value was compared to a distribution of mean global Fst values from 25 SNPs sampled at random 10,000 times from the empirical distribution. We found that disease-associated SNPs are not more differentiated than random markers (*P *= 0.462, *P*_*cor *_= 0.500). This analysis was repeated for groups of SNPs associated with each of the diseases listed in Table 1. In no case were the disease-associated SNPs more differentiated than expected at random (*P *and *P*_*cor *_> 0.3 in every case).

Global Fst provides a rough measure of the magnitude of allele frequency differentiation worldwide, but local positive selection acting at finer geographical scales will likely remain undetected using this measure. To examine the patterns of population differentiation at a more refined geographical scale, we calculated Fst for every pairwise comparison among the 53 populations and 7 geographic regions to produce 53 × 53 and 7 × 7 Fst matrices, respectively. Each Fst value was then compared to the corresponding empirical distribution of Fst values to generate a *P *value without correction for allele frequency.

**Figure 4:**
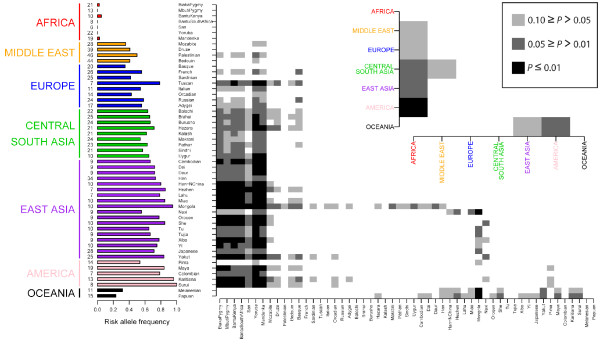
**Worldwide risk allele frequencies and population differentiation for rs10761659, a SNP associated with Crohn's disease.** The vertical bar chart displays risk allele frequencies in each of the populations represented in the CEPH-HGDP panel with sample sizes in number of individuals on the left. The shaded boxes in the 53 × 53 and 7 × 7 matrices show which pairwise Fst values are significant compared to the empirical distribution at three *P *value thresholds (see the boxed-in *P *value legend).

Figure 4 shows risk allele frequencies across populations and the two Fst matrices for the most highly-differentiated disease SNP rs10761659, a variant associated with Crohn's disease. Allele frequency and Fst estimates for populations with small sample sizes and/or missing genotypes may be unreliable and sample size is therefore also included in Figure 4. For rs10761659, the risk allele is rare in Africa but is found at high frequency in most non-African populations. The degree of differentiation at this SNP is unusually high compared to the empirical distribution as indicated by the low P values (i.e. dark boxes in Figure 4) in population pairwise comparisons between Africans and most non-African populations. We have produced similar plots for all 25 disease-associated SNPs for visual inspection (Additional file 3).

## Discussion

The extent to which the CDCV hypothesis is applicable across human populations depends in part on the extent to which common risk alleles identified in one population are also common in other populations. The majority of disease association studies are conducted using case-control cohorts of European ancestry. The degree to which associations established in these studies can be extended to other populations remains an open question. In addition, it remains unclear how often differences in risk allele frequencies between populations are due to the action of local positive selection. The present study takes a first step in addressing these issues by quantifying the degree of allele frequency differentiation between worldwide populations for 25 SNPs associated with 6 common complex diseases.

Many of the disease-associated SNPs studied here show substantial heterogeneity in allele frequencies across human populations (Figure 1). In some cases, risk allele frequencies remain generally low or high across all 53 populations. However, in several cases risk allele frequencies vary across a large portion of the allele frequency spectrum. Maximum allele frequency differences between any 2 populations ranged from 0.10 to 1.0 across SNPs with a mean of 0.65 (Figure 2). For 7 of the 25 SNPs, the maximum allele frequency difference between any 2 populations was > 0.75. Thus, some risk alleles are found at substantially different frequencies between populations.

To further quantify the allele frequency differences between populations for the disease-associated SNPs, we compared Fst values for the disease-associated SNPs to an empirical Fst distribution generated from 2750 random markers genotyped in the same samples. The average global Fst of the disease-associated SNPs is not unusually high compared to the empirical global Fst distribution. This is also the case when global Fst values were averaged across SNPs in each disease category. Thus, disease-associated SNPs do not show more population differentiation than random SNPs, in agreement with a previous study that examined a different set of disease-associated markers in a more limited set of populations [24].

Although disease-associated SNPs do not show high Fst as a set, individual disease-associated SNPs may be unusually differentiated. Previous studies have identified disease-associated loci that show evidence of local positive selection in the form of unusually large allele frequency differences between populations [14,15,17,25,51-53]. In some cases it is the protective allele [17,53], and in others the risk allele [15], which appears to have been driven to high frequency by positive selection. Several of the disease-associated SNPs studied here show considerable worldwide population differentiation and have global Fst values within the top 10% of the empirical global Fst distribution (Figure 3). At a more refined geographical scale, the patterns of population differentiation are extremely varied across SNPs and many population-pairwise Fst values lie within the top 5% and even the top 1% of the empirical distribution (see Additional file 3). For example, the risk allele at SNP rs10761659 is absent in some African populations and is near or at fixation in a number of populations outside of Africa. The global Fst value for this SNP lies within the top 5% of the empirical distribution (Figure 3) and most population pairwise comparisons between Africans and non-Africans are highly significant (Figure 4). A type 1 diabetes-associated SNP, rs11171739, also shows high levels of differentiation between Africans and non-Africans, but in this case the risk allele is near fixation in Africans but is at low to intermediate frequency elsewhere in the world (Additional file 3). There are also cases in which a risk allele frequency is unusually high or low in only one or a few populations. For example, the risk allele at rs564398, a SNP associated with type 2 diabetes, is found at unusually low frequencies only in the Kalash of Pakistan and in Melanesians (Additional file 3). These SNPs may therefore turn out to have been the targets of local positive selection. However, evidence for selection based on single marker Fst values should be interpreted with caution [54]. A more in-depth investigation of the patterns of genetic variation in and around these loci and their effects on the phenotype is required before conclusions can be confidently drawn.

Regardless of whether large risk allele frequency differences between populations are the result of selection or genetic drift, these data provide several useful insights. First, it is reasonable to assume that, if a risk allele is fixed, absent, or close to either, it does not contribute to disease risk variation within that population. Thus, assuming that the risk conferred by these alleles is constant across populations (as may be the case for risk alleles found in genes related to fundamental biological activity, e.g. cyclin dependent kinase function and T2D/CAD risk), our data suggest that the CDCV model does not necessarily extend across populations since risk alleles discovered in a European population are sometimes absent, fixed or found at extremely low or high frequencies in other populations.

Second, combining evidence of selection and association may enhance power to identify genotype-phenotype relationships: a SNP with a large difference in risk allele frequency between populations is a strong candidate to explain large differences in disease prevalence between populations [15,18]. However, despite the pattern observed for the Crohn's disease-associated SNP rs10761659 (Figure 4), there is no strong evidence to suggest that the risk of developing Crohn's disease differs dramatically between individuals of African and European ancestry [55]. Future studies are required to determine the extent to which differences in risk allele frequencies between populations predict disease prevalence differences between populations.

Finally, power estimates for disease association studies rely on estimates of the risk allele frequency in a population [56]. Inaccurate risk allele frequency estimates can result in overestimates of power and, consequently, in underpowered studies [57,58]. Thus, these data can aid in the design of future association studies in populations for which allele frequency data are scarce.

Some of the risk alleles studied here may not be disease causing, but instead may be in linkage disequilibrium (LD) with the disease causing allele. Although recombination hotspot locations are generally shared across human populations and there is substantial conservation of haplotype structure worldwide [49,59], the extent of LD can vary markedly across populations [60-63]. Because LD breaks down differently in different populations, the risk alleles studied here may not be associated with disease across all human populations. Our analyses assume that the degree of LD between the genotyped risk allele and the true causal allele is conserved across populations. Our interpretations should be considered in light of this caveat.

Disease-association studies have primarily made use of case-control cohorts of European ancestry. Studies of worldwide patterns of genetic variation in disease-associated genes are essential to determine how transferable disease-gene associations are from one population to another. Moreover, disease-association studies in diverse populations are required in order to determine whether different alleles are responsible for disease prevalence in different populations. A strong focus on the genetics of disease in humans worldwide is an important step in addressing large disparities in the quality of health care between human populations.

## Conclusion

Disease-associated SNPs do not differ in frequency more between human populations than random SNPs in the genome. This suggests that positive local selection has not had a strong effect on the frequencies of risk alleles in general. Individually, however, several disease-associated SNPs do show evidence of positive local selection. Regardless of whether the observed differences are due to drift or selection, worldwide variation in risk allele frequencies is considerable. Future studies are required to determine the extent to which this variation is responsible for differences in disease prevalence between populations.

## Competing interests

The authors declare that they have no competing interests.

## Authors' contributions

SM, JB, MS and NT designed the study; SM and DD performed statistical analyses; SM, DD, JB, MS and NT wrote the manuscript.

## Pre-publication history

The pre-publication history for this paper can be accessed here:



## Supplementary Material

Additional file 1 Correlation between minor allele frequency and global Fst for 2750 markers typed in 927 individuals from the CEPH-HGDP panel. Click here for file

Additional file 2 Global Fst density distributions for 2750 markers typed in the 927 individuals from the CEPH-HGDP panel divided into 5 bins according to minor allele frequency. Click here for file

Additional file 3 Worldwide risk allele frequencies and population differentiation for 25 disease-associated SNPs. The dbSNP ID and disease for each SNP is found at the top of each figure. Risk allele frequencies were calculated using data from 952 individuals from the CEPH-HGDP panel and are displayed in the vertical bar chart with sample size in number of individuals to the left. Pairwise Fst values for the 53 × 53 population matrix and the 7 × 7 geographical region matrix were calculated using data from the same 927 individuals who were used to generate the empirical distribution. Each square in the 53 × 53 and 7 × 7 matrices represents a pairwise Fst comparison between populations and geographic regions, respectively. The shaded boxes in the matrices indicate which pairwise Fst values are significant compared to the empirical distribution at three *P *value thresholds (see the boxed-in *P *value legend of Figure 2). Click here for file
