# Supplementary figures and images for: Worldwide population differentiation at disease-associated SNPs
Source: BMC Med Genomics. 2008 Jun 4;1:22. doi: 10.1186/1755-8794-1-22 (PMC2440747; doi:10.1186/1755-8794-1-22)

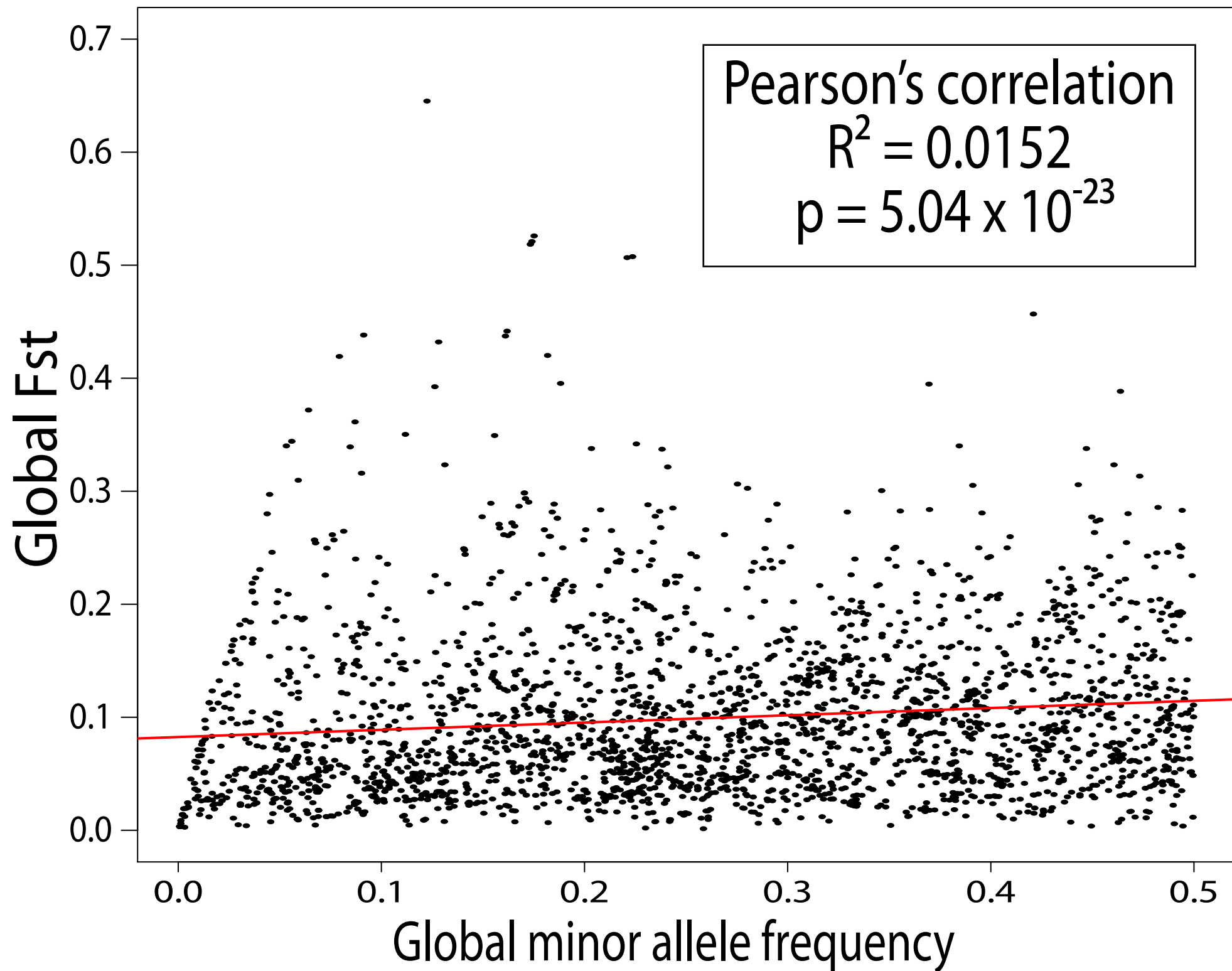

Supplement: Additional file 1 — Correlation between minor allele frequency and global Fst for 2750 markers typed in 927 individuals from the CEPH-HGDP panel. [file 1755-8794-1-22-S1.pdf]

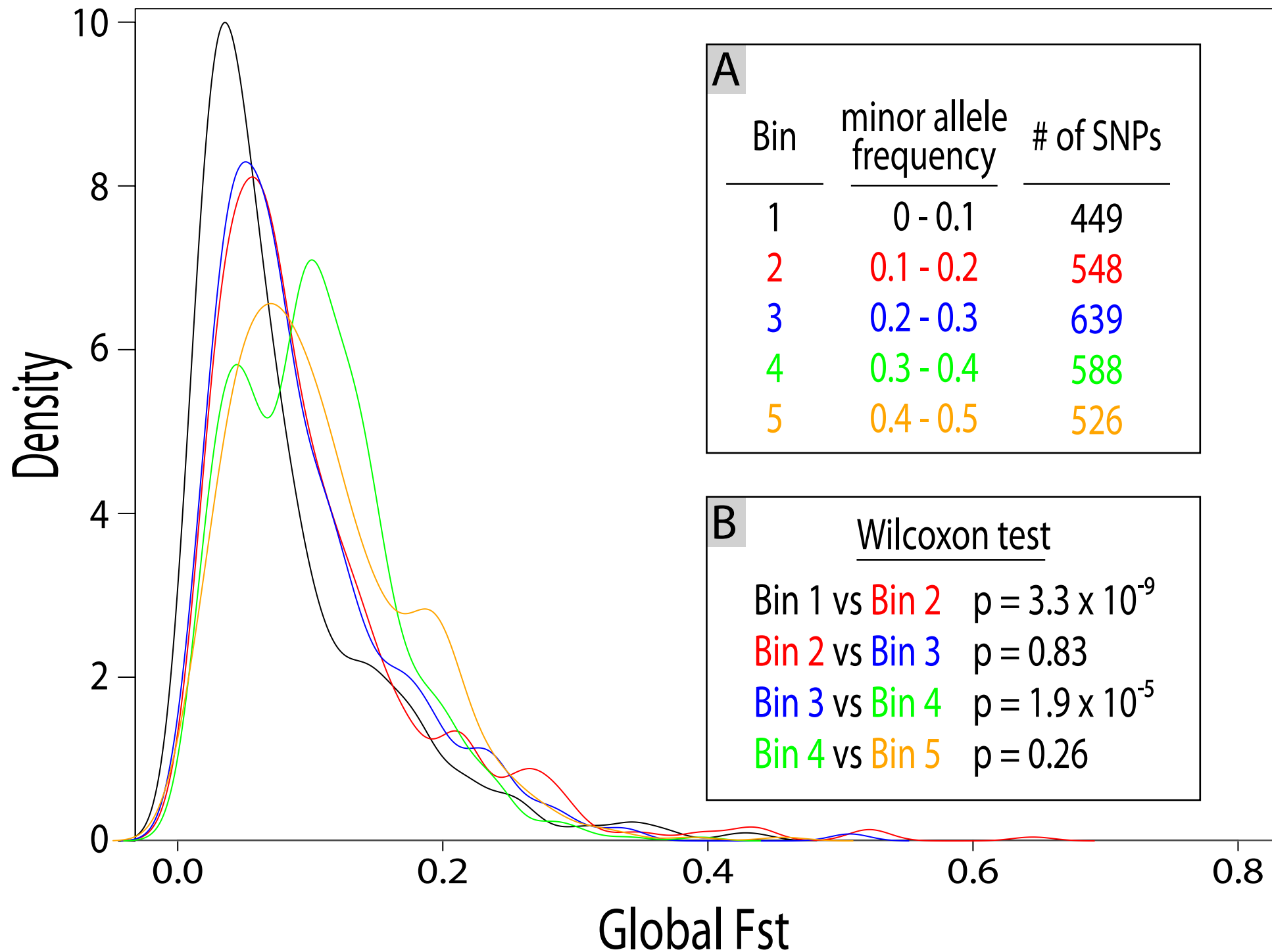

Supplement: Additional file 2 — Global Fst density distributions for 2750 markers typed in the 927 individuals from the CEPH-HGDP panel divided into 5 bins according to minor allele frequency. [file 1755-8794-1-22-S2.pdf]
