# Supplementary material for: Worldwide population differentiation at disease-associated SNPs
Source: BMC Med Genomics. 2008 Jun 4;1:22. doi: 10.1186/1755-8794-1-22 (PMC2440747; doi:10.1186/1755-8794-1-22)
Supplement: Additional file 3 — Worldwide risk allele frequencies and population differentiation for 25 disease-associated SNPs. The dbSNP ID and disease for each SNP is found at the top of each figure. Risk allele frequencies were calculated using data from 952 individuals from the CEPH-HGDP panel and are displayed in the vertical bar chart with sample size in number of individuals to the left. Pairwise Fst values for the 53 × 53 population matrix and the 7 × 7 geographical region matrix were calculated using data from the same 927 individuals who were used to generate the empirical distribution. Each square in the 53 × 53 and 7 × 7 matrices represents a pairwise Fst comparison between populations and geographic regions, respectively. The shaded boxes in the matrices indicate which pairwise Fst values are significant compared to the empirical distribution at three P value thresholds (see the boxed-in P value legend of Figure 2). [file 1755-8794-1-22-S3.pdf]

rs1801282 T2D

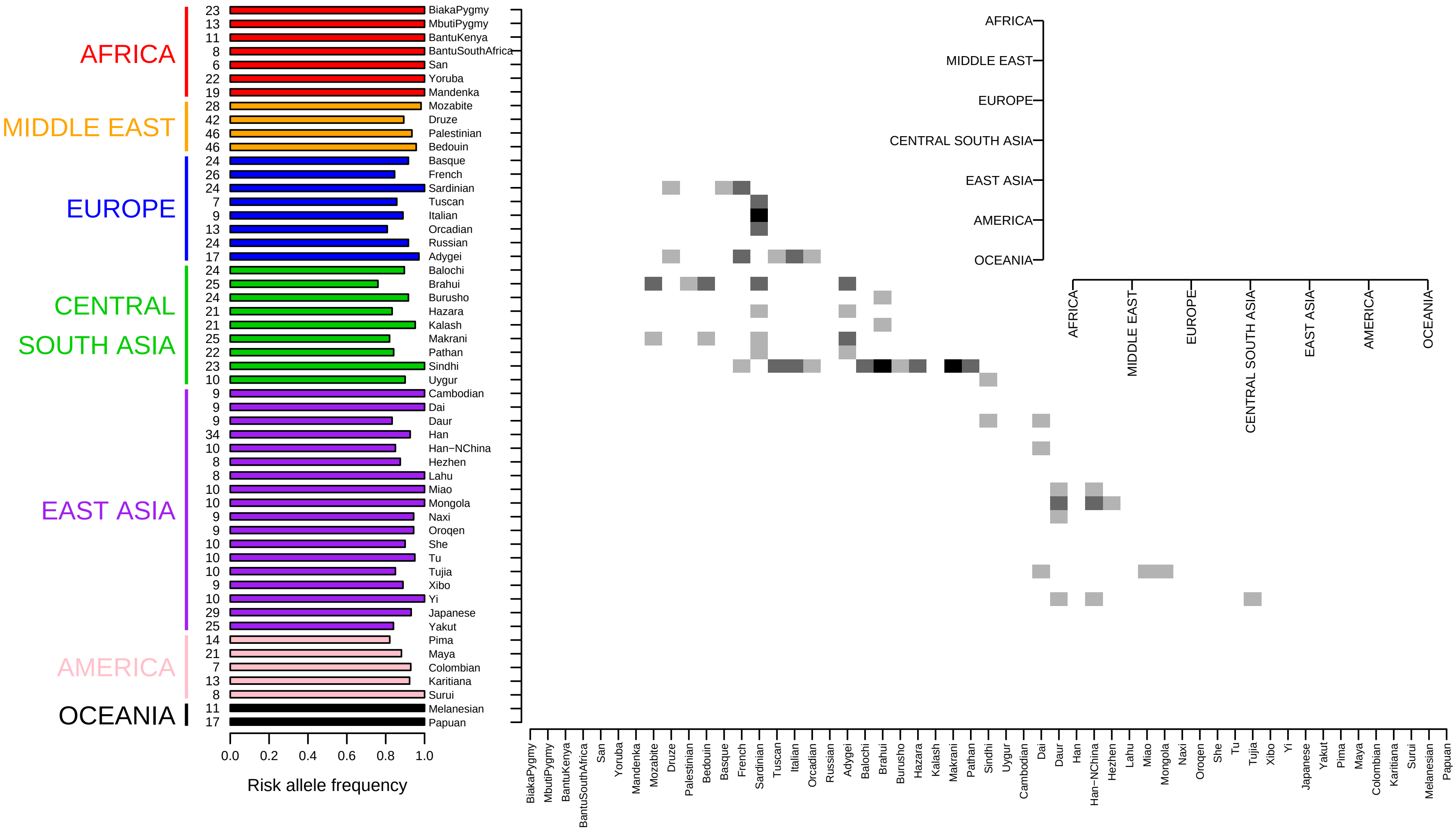

rs9939609 T2D/OB

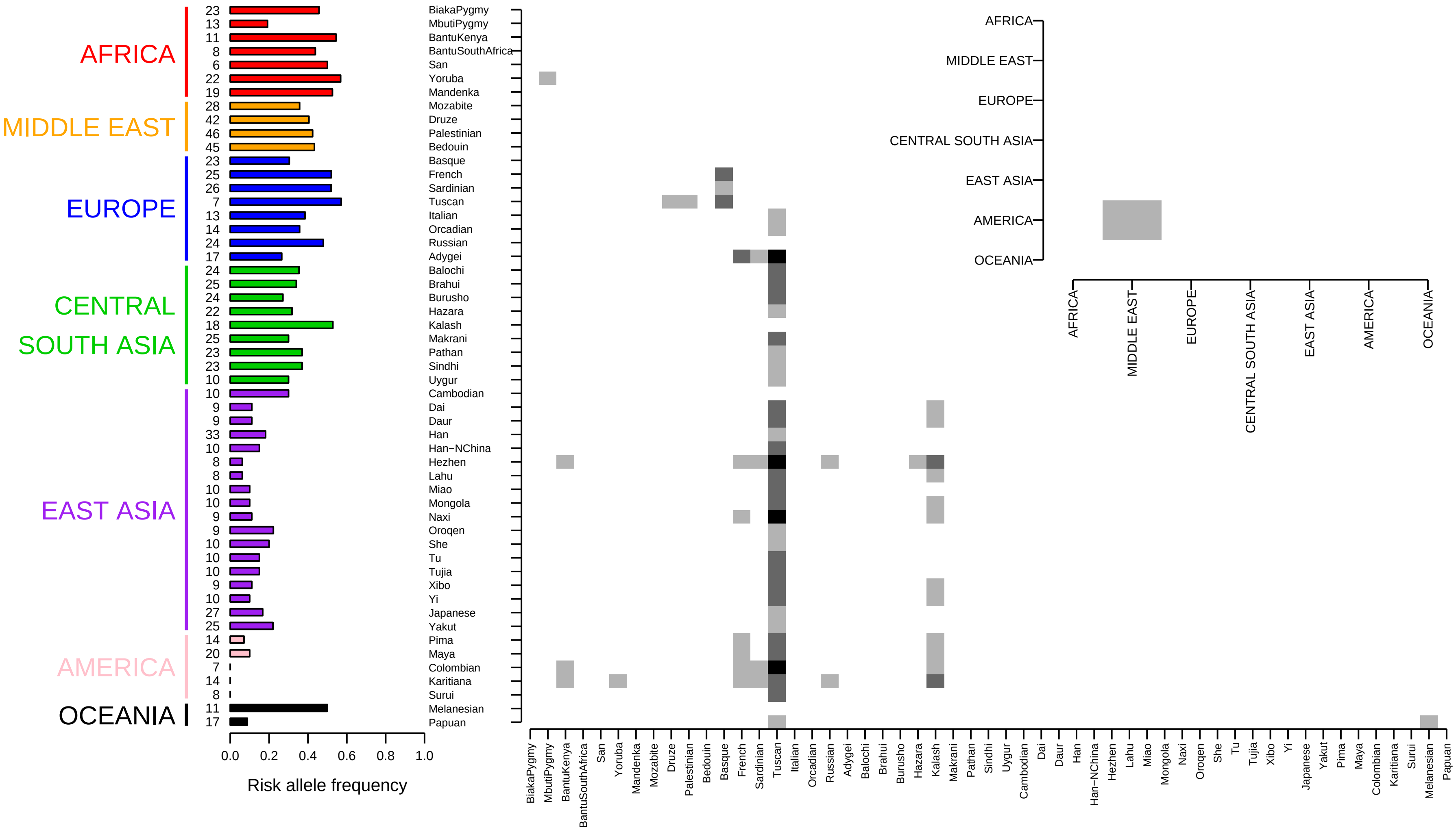

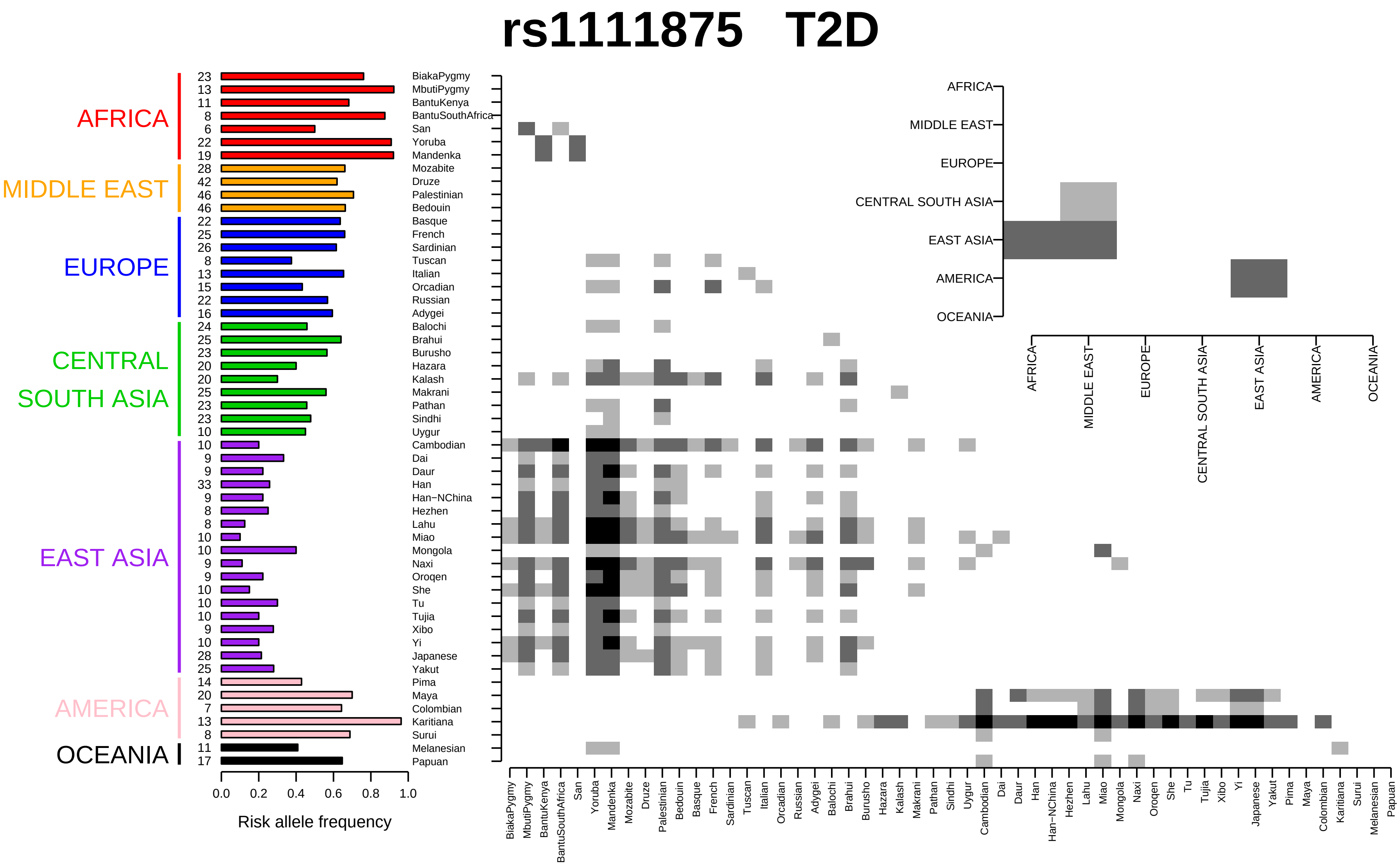
rs1111875 T2D
AFRICA
MIDDLE EAST
EUROPE
CENTRAL SOUTH ASIA
EAST ASIA
AMERICA
OCEANIA
23 BiakaPygmy
13 MbutiPygmy
11 BantuKenya
8 BantuSouthAfrica
6 San
22 Yoruba
19 Mandenka
28 Mozabite
42 Druze
46 Palestinian
46 Bedouin
22 Basque
25 French
26 Sardinian
8 Tuscan
13 Italian
15 Orcadian
22 Russian
16 Adygei
24 Balochi
25 Brahui
23 Burusho
20 Hazara
20 Kalash
25 Makrani
23 Pathan
23 Sindhi
10 Uygur
10 Cambodian
9 Dai
9 Daur
33 Han
9 Han−NChina
8 Hezhen
8 Lahu
10 Miao
10 Mongola
9 Naxi
9 Oroqen
10 She
10 Tu
10 Tujia
9 Xibo
10 Yi
28 Japanese
25 Yakut
14 Pima
20 Maya
7 Colombian
13 Karitiana
8 Surui
11 Melanesian
17 Papuan
0.0
0.2
0.4
0.6
0.8
1.0
Risk allele frequency

rs4402960 T2D

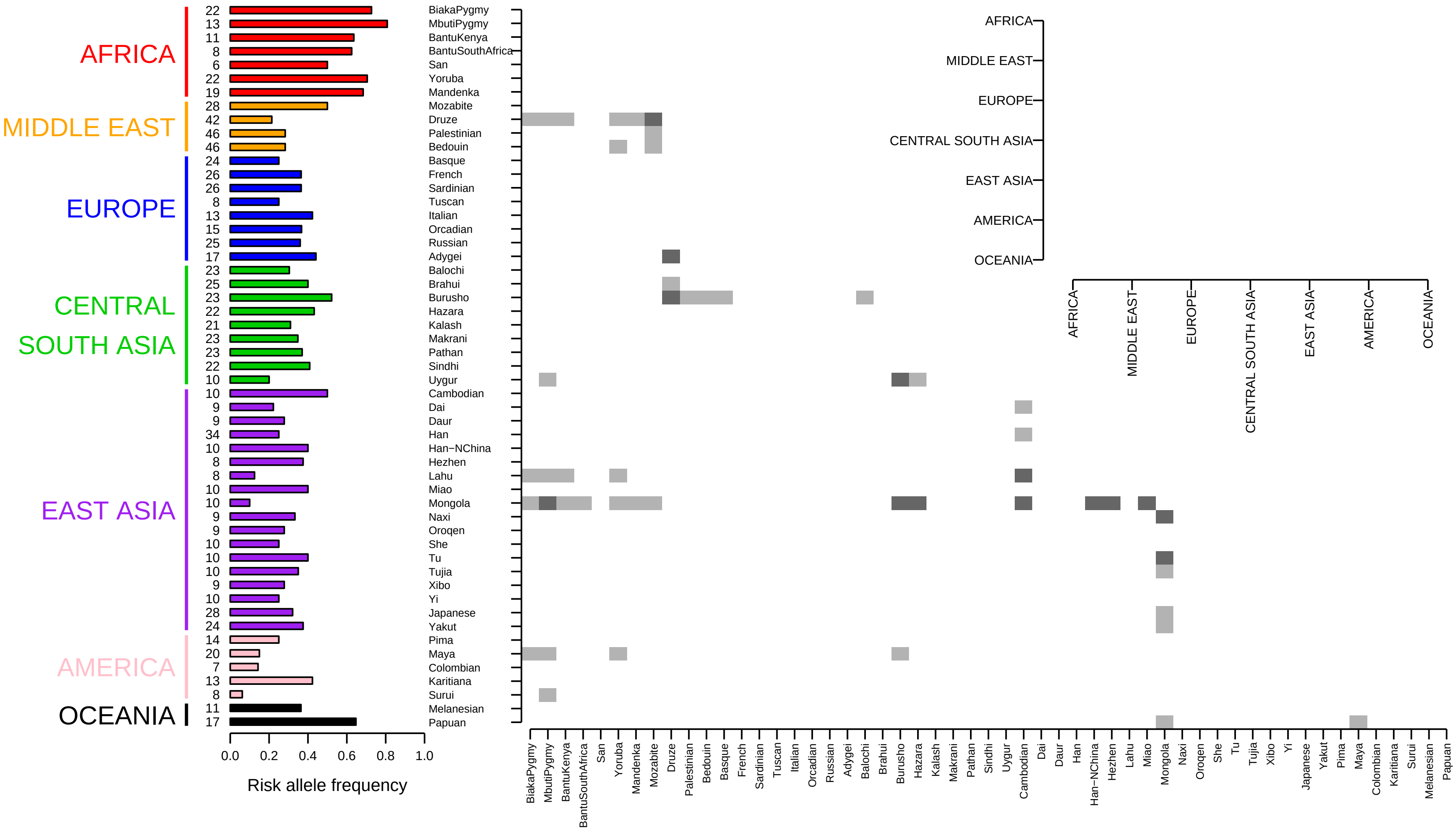

rs564398 T2D

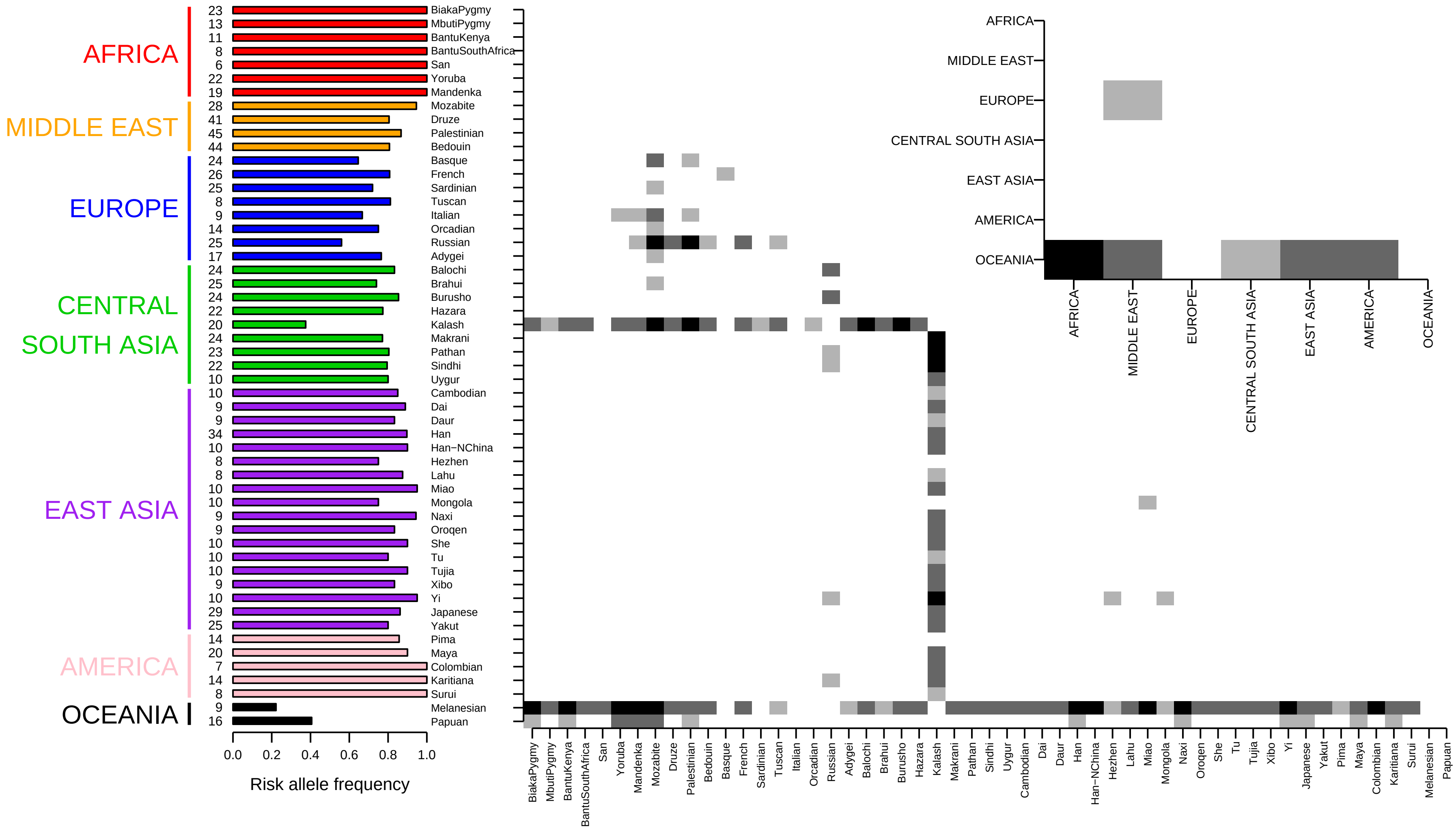

rs1333049 CAD

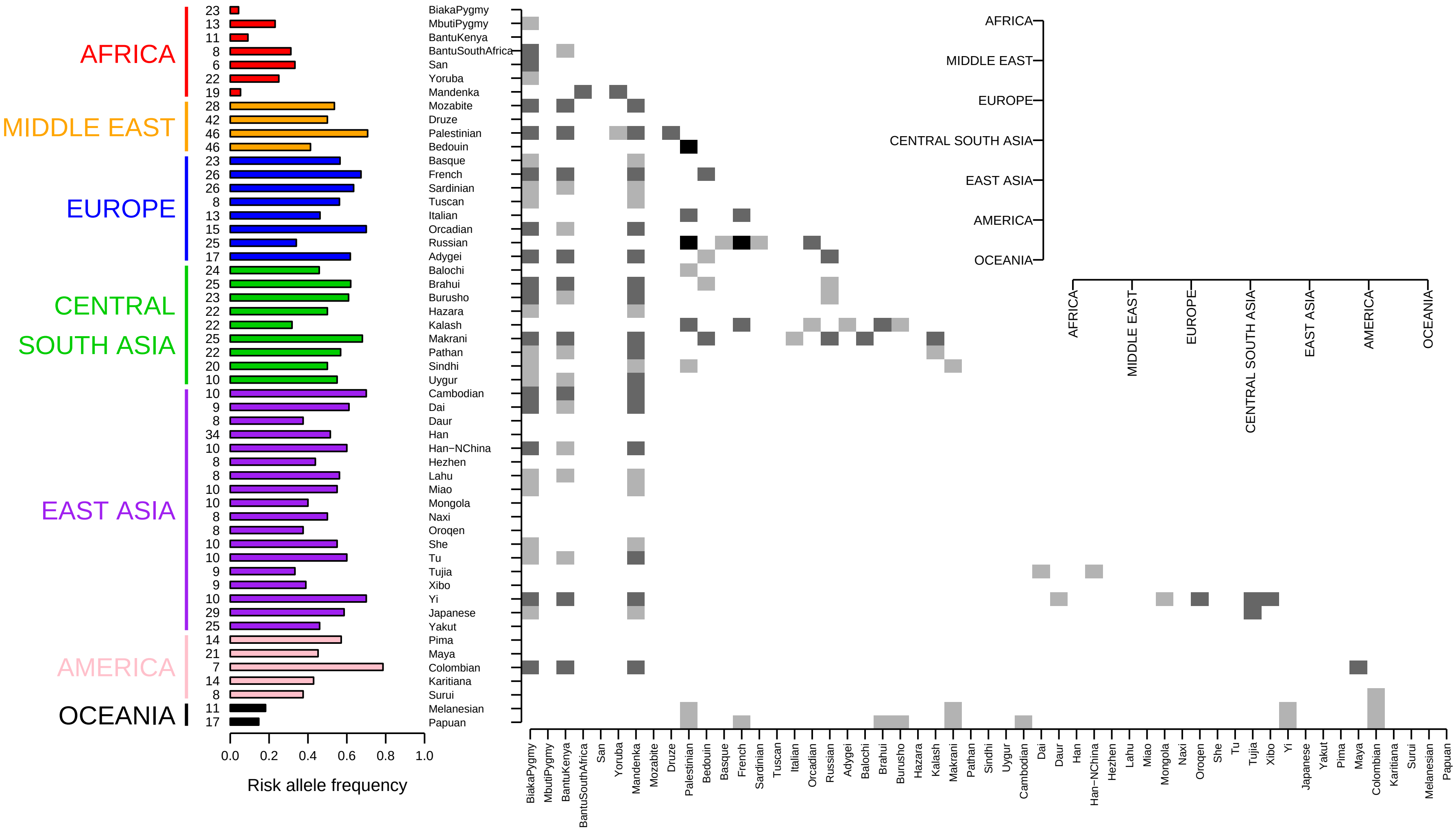

rs11805303 CD

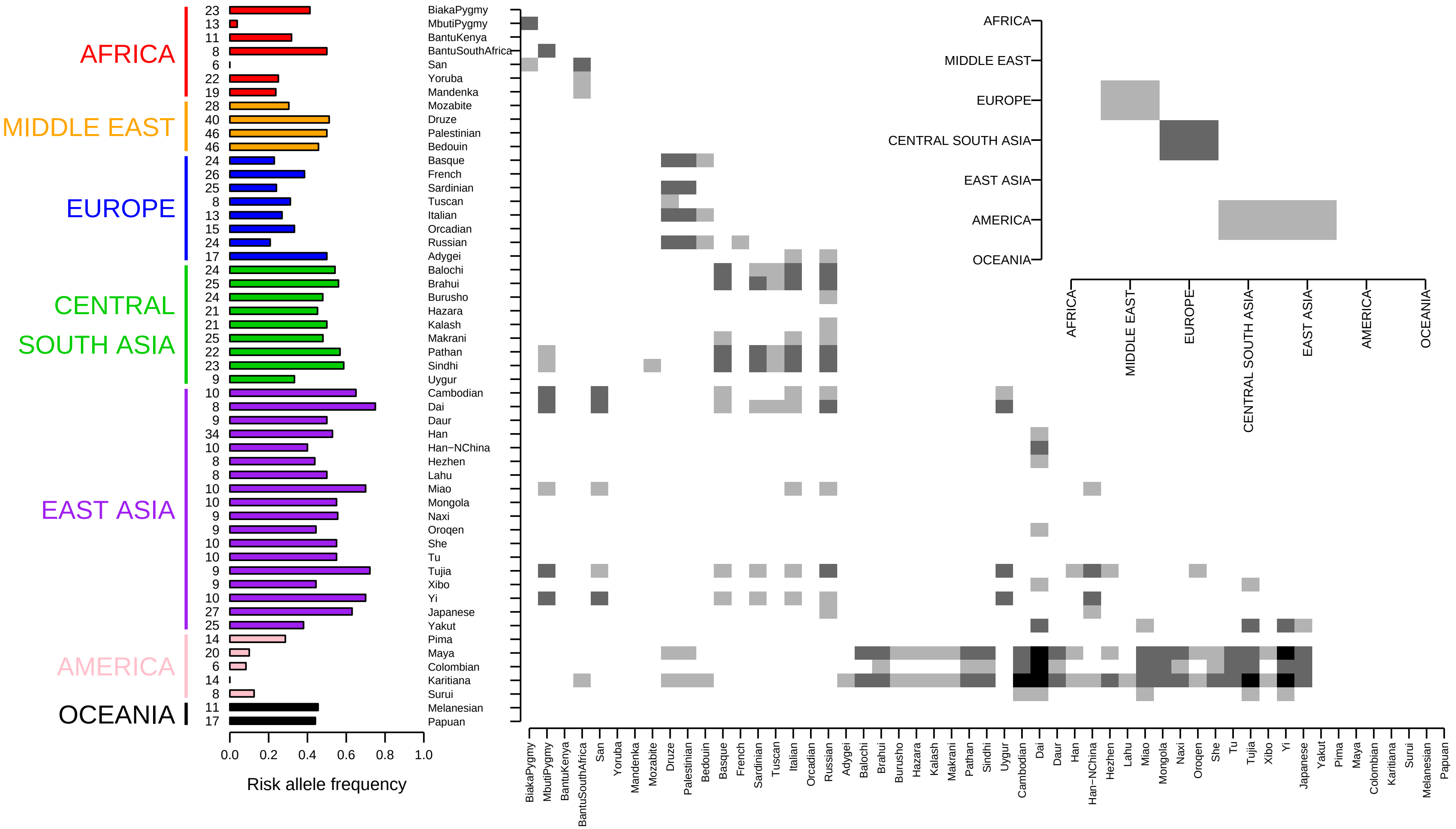

rs10210302 CD

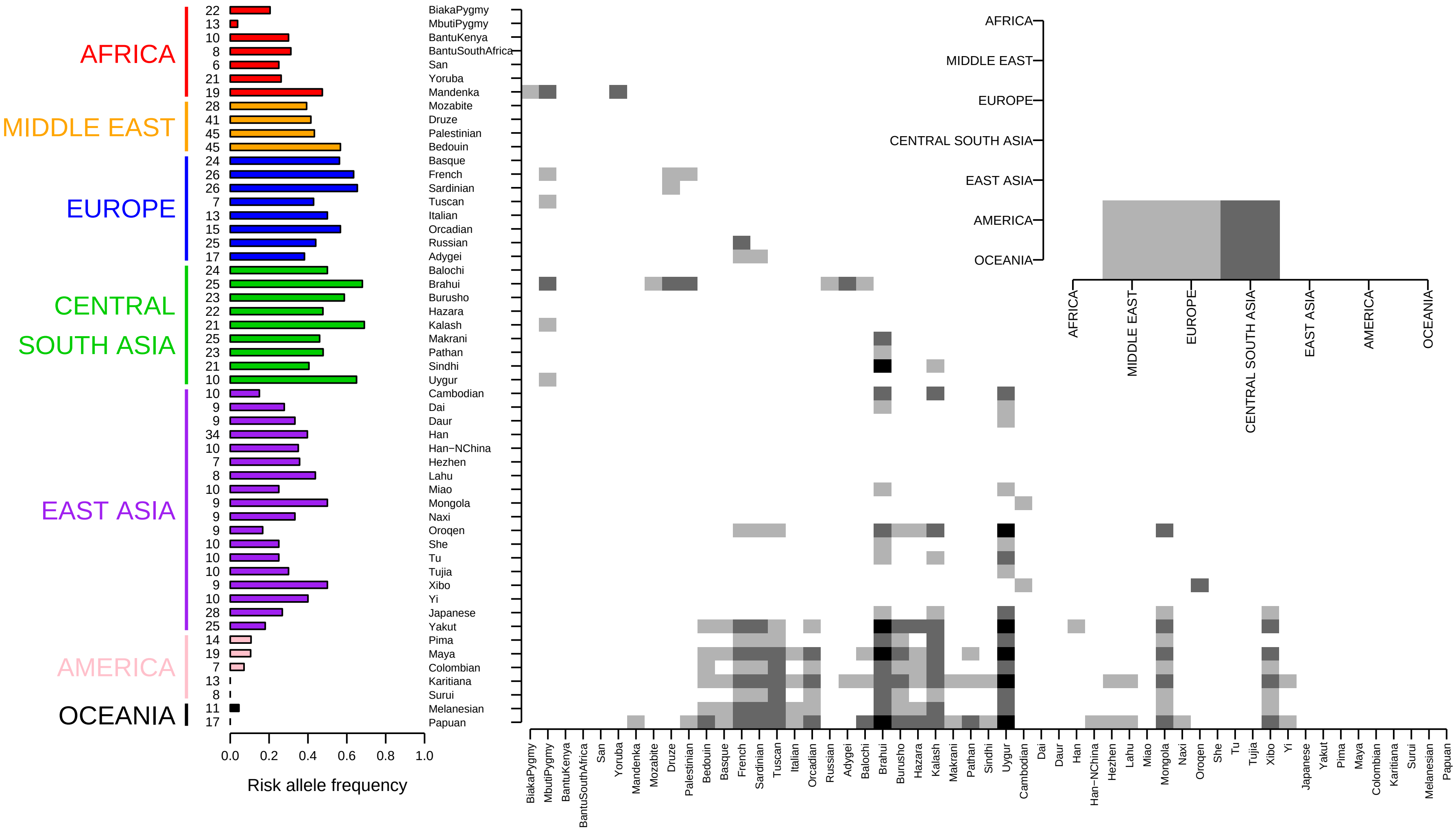

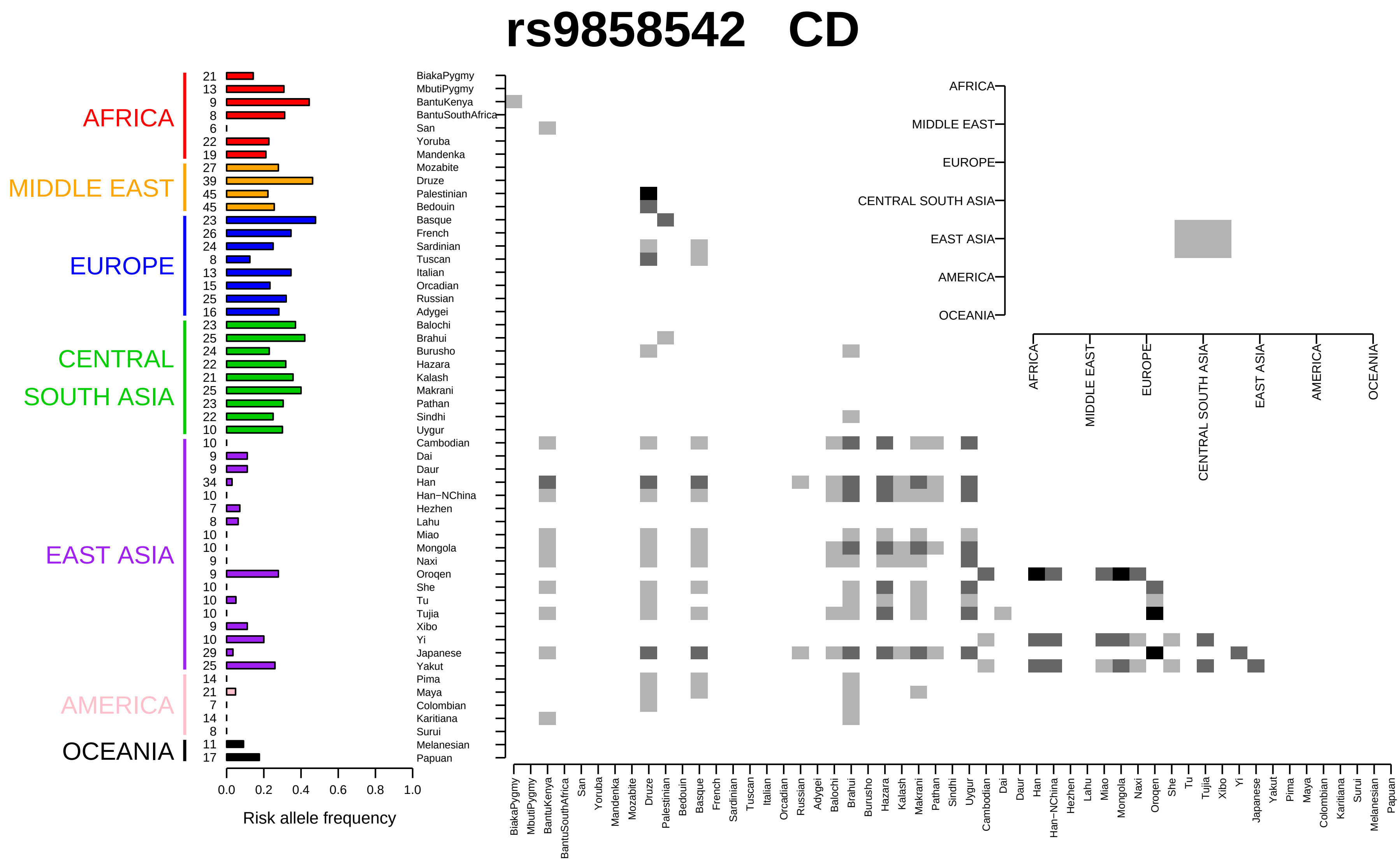

rs9858542 CD
AFRICA
MIDDLE EAST
EUROPE
CENTRAL SOUTH ASIA
EAST ASIA
AMERICA
OCEANIA
Risk allele frequency
0.0 0.2 0.4 0.6 0.8 1.0
BiakaPygmy MbutiPygmy BantuKenya BantuSouthAfrica San Yoruba Mandenka Mozabite Druze Palestinian Bedouin Basque French Sardinian Tuscan Italian Orcadian Russian Adygei Balochi Brahui Burusho Hazara Kalash Makrani Pathan Sindhi Uygur Cambodian Dai Daur Han Han−NChina Hezhen Lahu Miao Mongola Naxi Oroqen She Tu Tujia Xibo Yi Japanese Yakut Pima Maya Colombian Karitiana Surui Melanesian Papuan
21 13 9 8 6 22 19 27 39 45 45 23 26 24 8 13 15 25 16 23 25 24 22 21 25 23 22 10 10 9 9 34 10 7 8 10 10 9 9 10 10 10 9 10 29 25 14 21 7 14 8 11 17

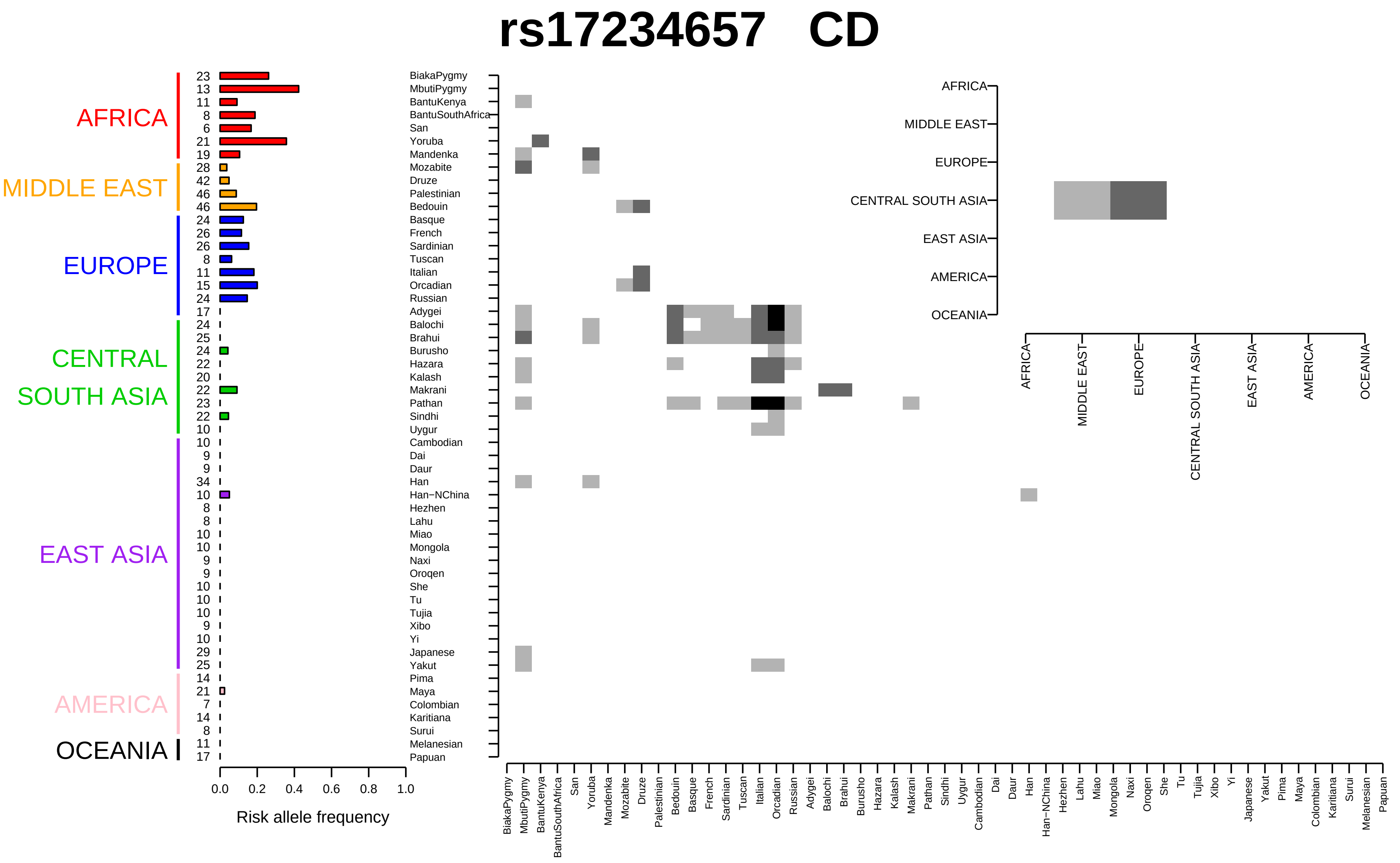
rs17234657 CD
AFRICA
MIDDLE EAST
EUROPE
CENTRAL SOUTH ASIA
EAST ASIA
AMERICA
OCEANIA
Risk allele frequency
BiakaPygmy
MbutiPygmy
BantuKenya
BantuSouthAfrica
San
Yoruba
Mandenka
Mozabite
Druze
Palestinian
Bedouin
Basque
French
Sardinian
Tuscan
Italian
Orcadian
Russian
Adygei
Balochi
Brahui
Burusho
Hazara
Kalash
Makrani
Pathan
Sindhi
Uygur
Cambodian
Dai
Daur
Han
Han−NChina
Hezhen
Lahu
Miao
Mongola
Naxi
Oroqen
She
Tu
Tujia
Xibo
Yi
Japanese
Yakut
Pima
Maya
Colombian
Karitiana
Surui
Melanesian
Papuan

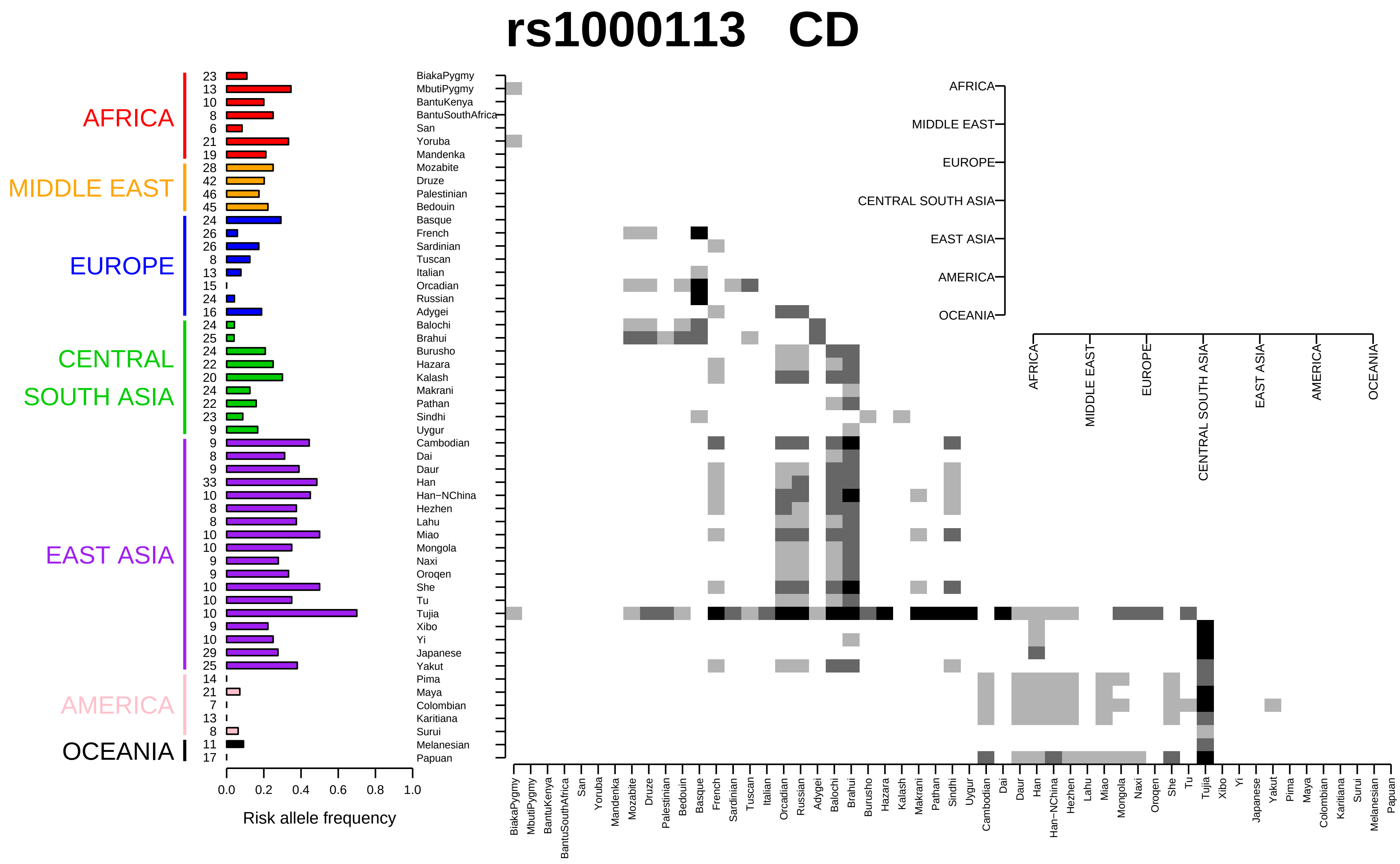

rs1000113 CD
AFRICA
MIDDLE EAST
EUROPE
CENTRAL SOUTH ASIA
EAST ASIA
AMERICA
OCEANIA
23 13 10 8 6 21 19 28 42 46 45 24 26 26 8 13 15 24 16 24 25 24 22 20 24 22 23 9 9 8 9 33 10 8 8 10 10 9 9 10 10 10 9 10 29 25 14 21 7 13 8 11 17
BiakaPygmy
MbutiPygmy
BantuKenya
BantuSouthAfrica
San
Yoruba
Mandenka
Mozabite
Druze
Palestinian
Bedouin
Basque
French
Sardinian
Tuscan
Italian
Orcadian
Russian
Adygei
Balochi
Brahui
Burusho
Hazara
Kalash
Makrani
Pathan
Sindhi
Uygur
Cambodian
Dai
Daur
Han
Han−NChina
Hezhen
Lahu
Miao
Mongola
Naxi
Oroqen
She
Tu
Tujia
Xibo
Yi
Japanese
Yakut
Pima
Maya
Colombian
Karitiana
Surui
Melanesian
Papuan
0.0 0.2 0.4 0.6 0.8 1.0
Risk allele frequency

rs10761659 CD

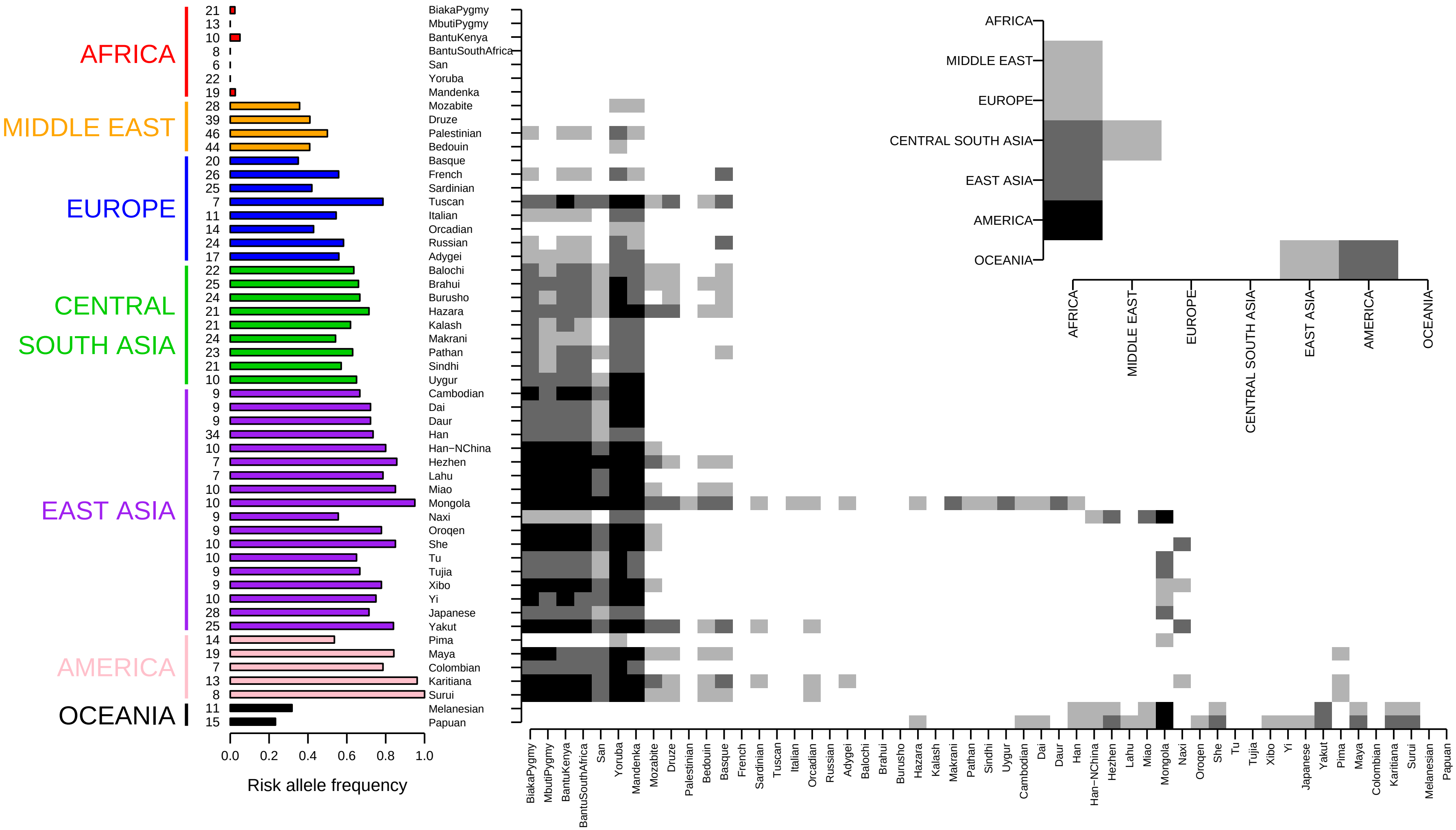

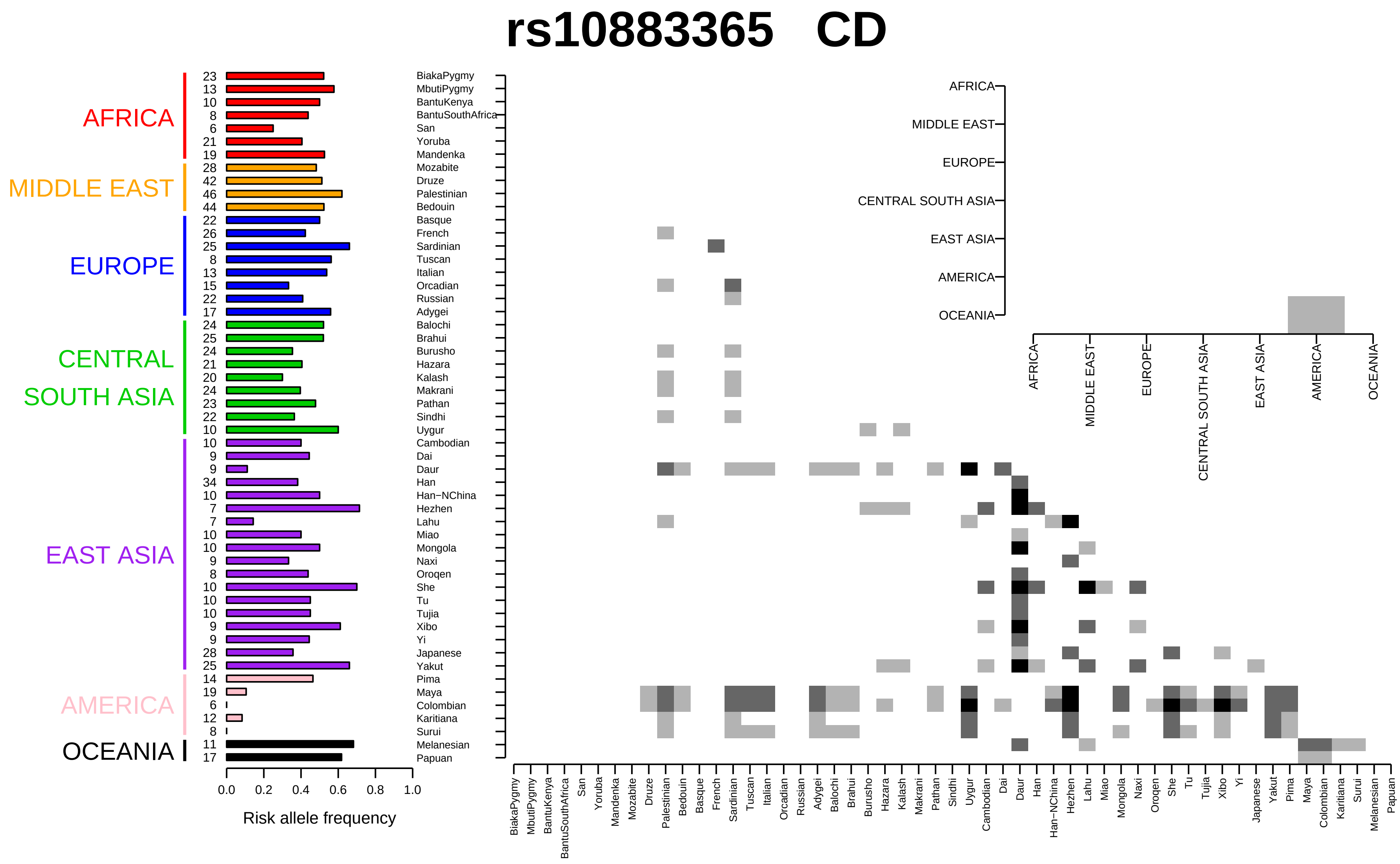

rs10883365 CD
AFRICA
MIDDLE EAST
EUROPE
CENTRAL SOUTH ASIA
EAST ASIA
AMERICA
OCEANIA
Risk allele frequency
0.0 0.2 0.4 0.6 0.8 1.0
23 BiakaPygmy
13 MbutiPygmy
10 BantuKenya
8 BantuSouthAfrica
6 San
21 Yoruba
19 Mandenka
28 Mozabite
42 Druze
46 Palestinian
44 Bedouin
22 Basque
26 French
25 Sardinian
8 Tuscan
13 Italian
15 Orcadian
22 Russian
17 Adygei
24 Balochi
25 Brahui
24 Burusho
21 Hazara
20 Kalash
24 Makrani
23 Pathan
22 Sindhi
10 Uygur
10 Cambodian
9 Dai
9 Daur
34 Han
10 Han−NChina
7 Hezhen
7 Lahu
10 Miao
10 Mongola
9 Naxi
8 Oroqen
10 She
10 Tu
10 Tujia
9 Xibo
9 Yi
28 Japanese
25 Yakut
14 Pima
19 Maya
6 Colombian
12 Karitiana
8 Surui
11 Melanesian
17 Papuan

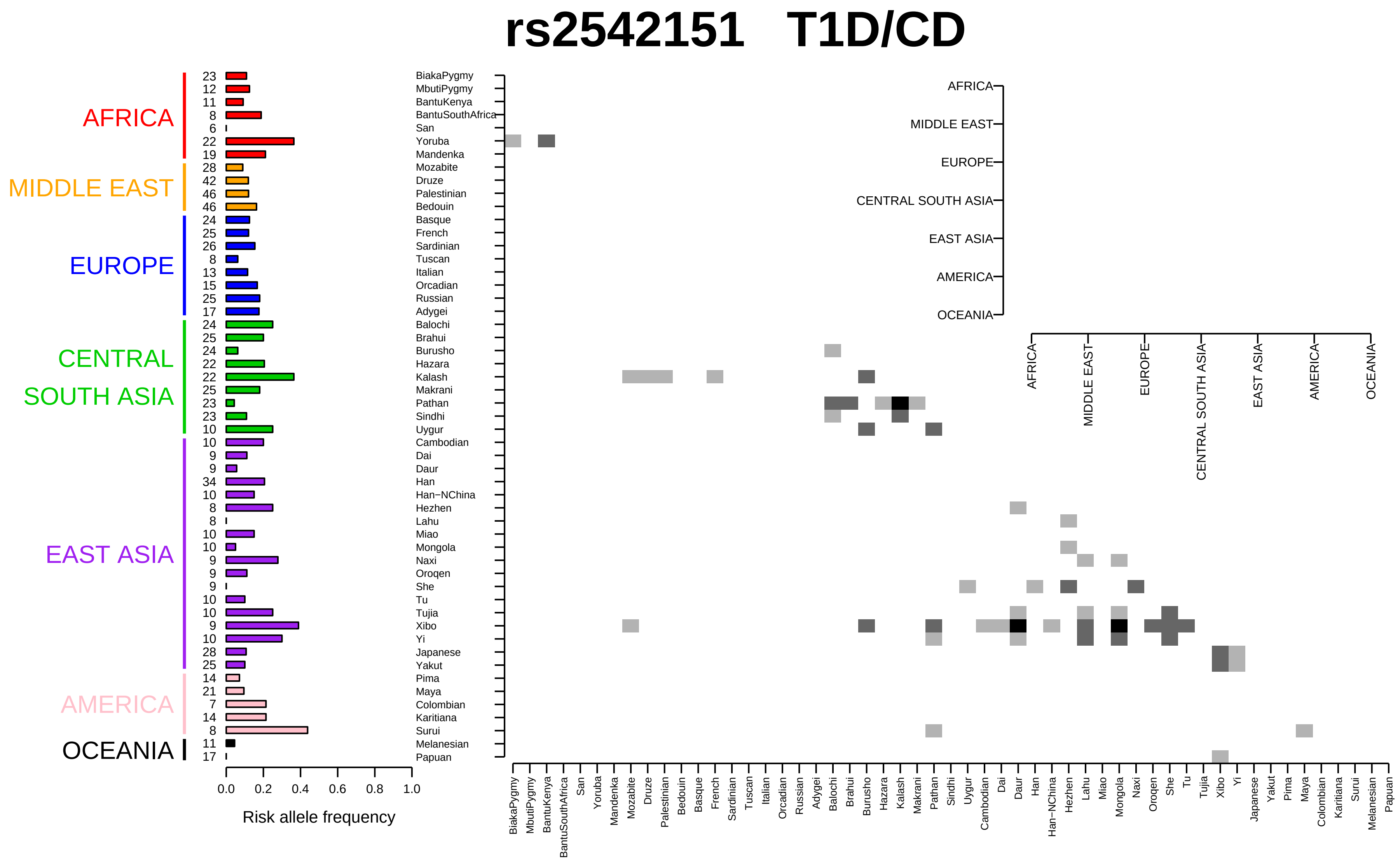
rs2542151 T1D/CD
AFRICA
MIDDLE EAST
EUROPE
CENTRAL SOUTH ASIA
EAST ASIA
AMERICA
OCEANIA
23 12 11 8 6 22 19 28 42 46 46 24 25 26 8 13 15 25 17 24 25 24 22 22 25 23 23 10 10 9 9 34 10 8 8 10 10 9 9 9 10 10 9 10 28 25 14 21 7 14 8 11 17
0.0 0.2 0.4 0.6 0.8 1.0
Risk allele frequency
BiakaPygmy
MbutiPygmy
BantuKenya
BantuSouthAfrica
San
Yoruba
Mandenka
Mozabite
Druze
Palestinian
Bedouin
Basque
French
Sardinian
Tuscan
Italian
Orcadian
Russian
Adygei
Balochi
Brahui
Burusho
Hazara
Kalash
Makrani
Pathan
Sindhi
Uygur
Cambodian
Dai
Daur
Han
Han−NChina
Hezhen
Lahu
Miao
Mongola
Naxi
Oroqen
She
Tu
Tujia
Xibo
Yi
Japanese
Yakut
Pima
Maya
Colombian
Karitiana
Surui
Melanesian
Papuan

rs10077785 CD

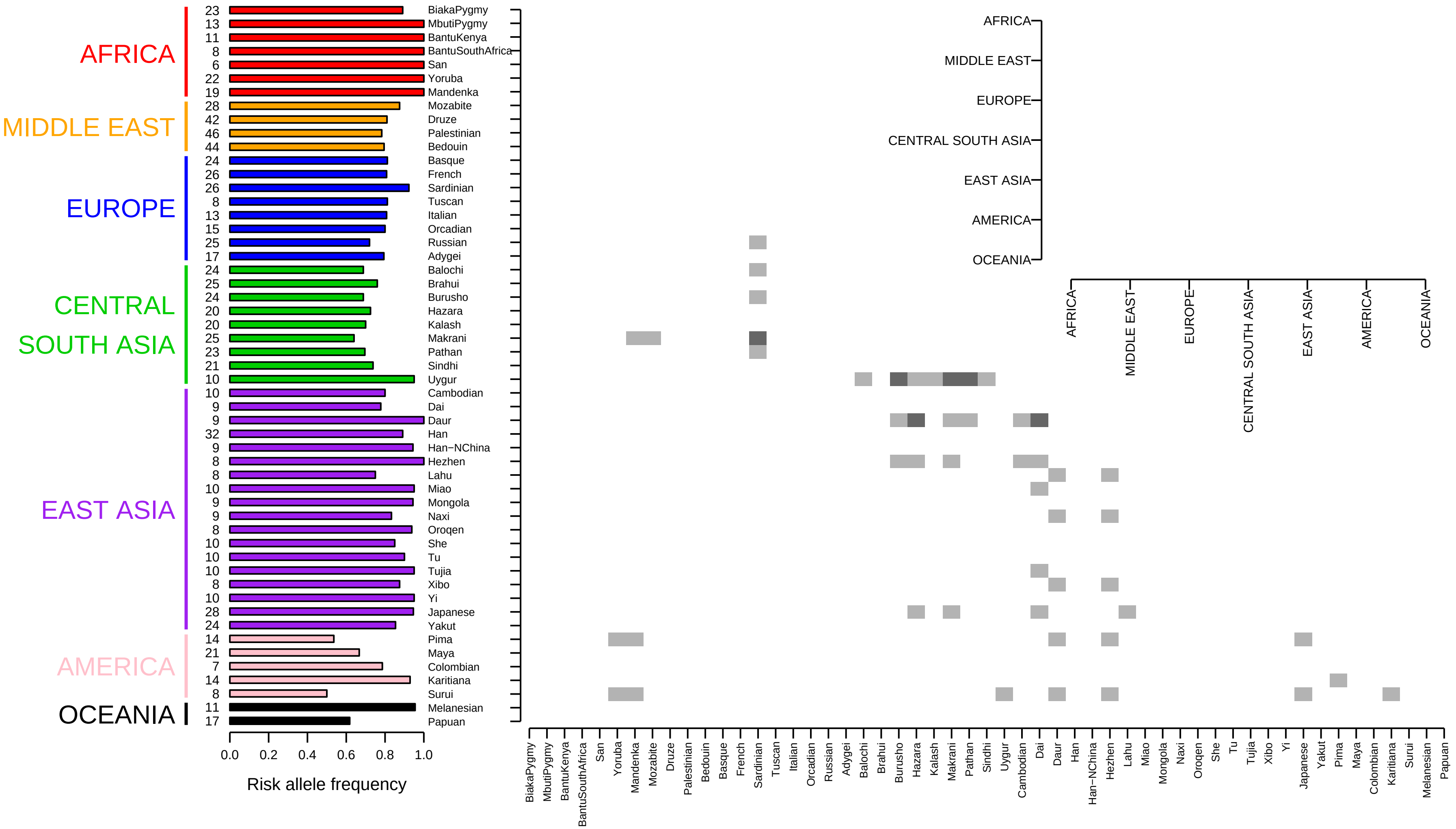

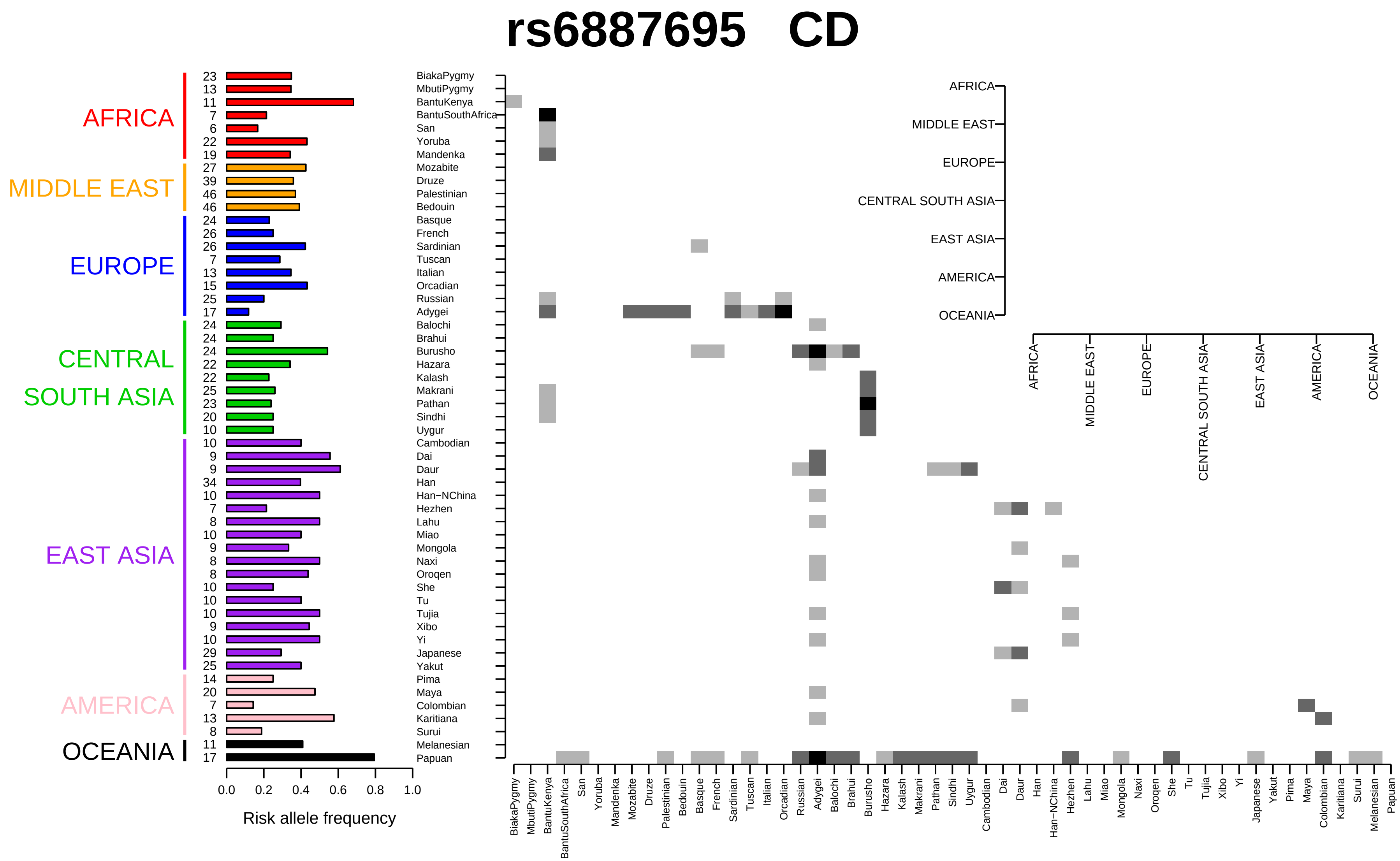
rs6887695 CD
AFRICA
MIDDLE EAST
EUROPE
CENTRAL SOUTH ASIA
EAST ASIA
AMERICA
OCEANIA
23 BiakaPygmy
13 MbutiPygmy
11 BantuKenya
7 BantuSouthAfrica
6 San
22 Yoruba
19 Mandenka
27 Mozabite
39 Druze
46 Palestinian
46 Bedouin
24 Basque
26 French
26 Sardinian
7 Tuscan
13 Italian
15 Orcadian
25 Russian
17 Adygei
24 Balochi
24 Brahui
24 Burusho
22 Hazara
22 Kalash
25 Makrani
23 Pathan
20 Sindhi
10 Uygur
10 Cambodian
9 Dai
9 Daur
34 Han
10 Han−NChina
7 Hezhen
8 Lahu
10 Miao
9 Mongola
8 Naxi
8 Oroqen
10 She
10 Tu
10 Tujia
9 Xibo
10 Yi
29 Japanese
25 Yakut
14 Pima
20 Maya
7 Colombian
13 Karitiana
8 Surui
11 Melanesian
17 Papuan
0.0 0.2 0.4 0.6 0.8 1.0
Risk allele frequency

rs6679677 RA/T1D

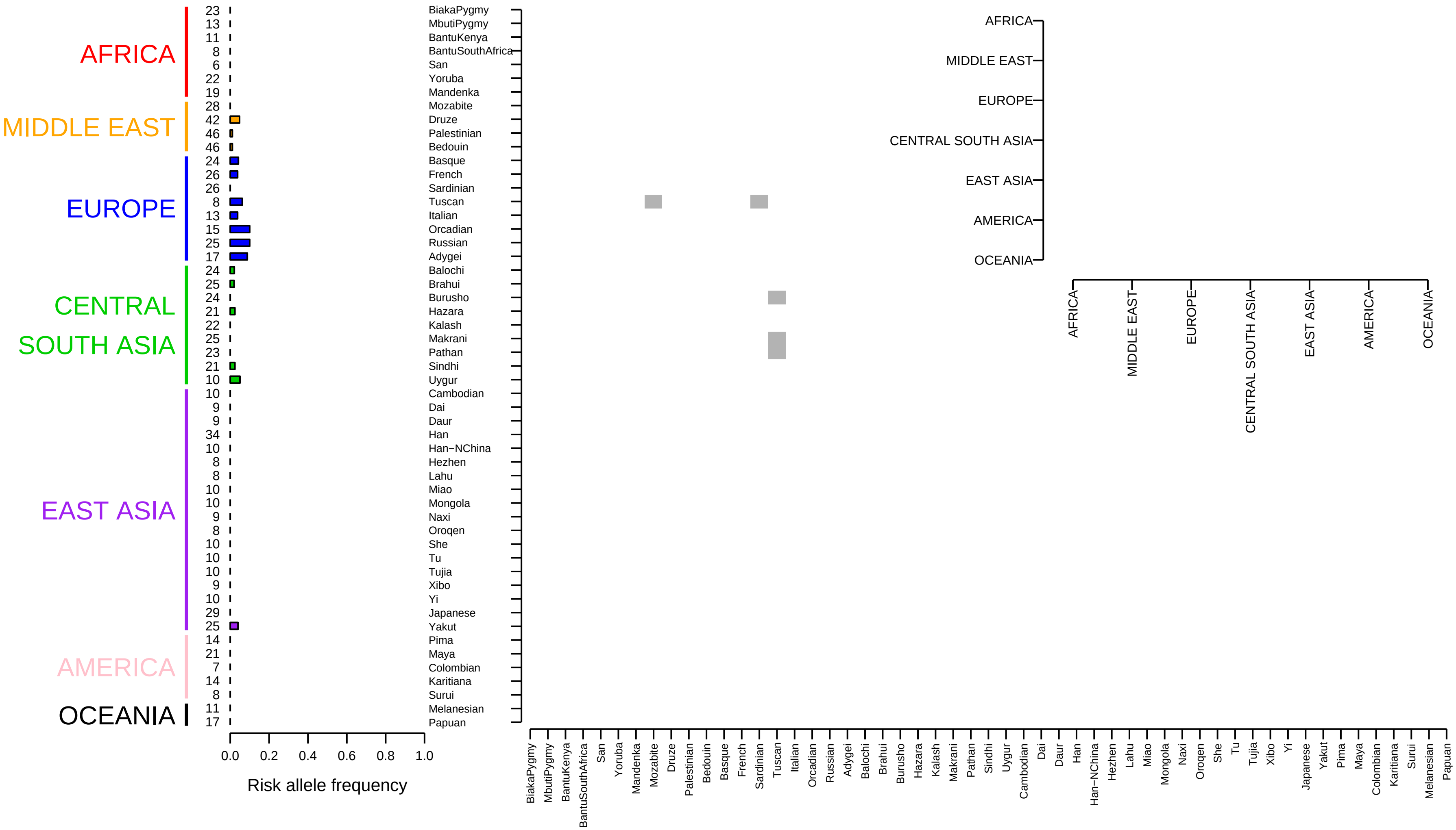

rs17696736 T1D

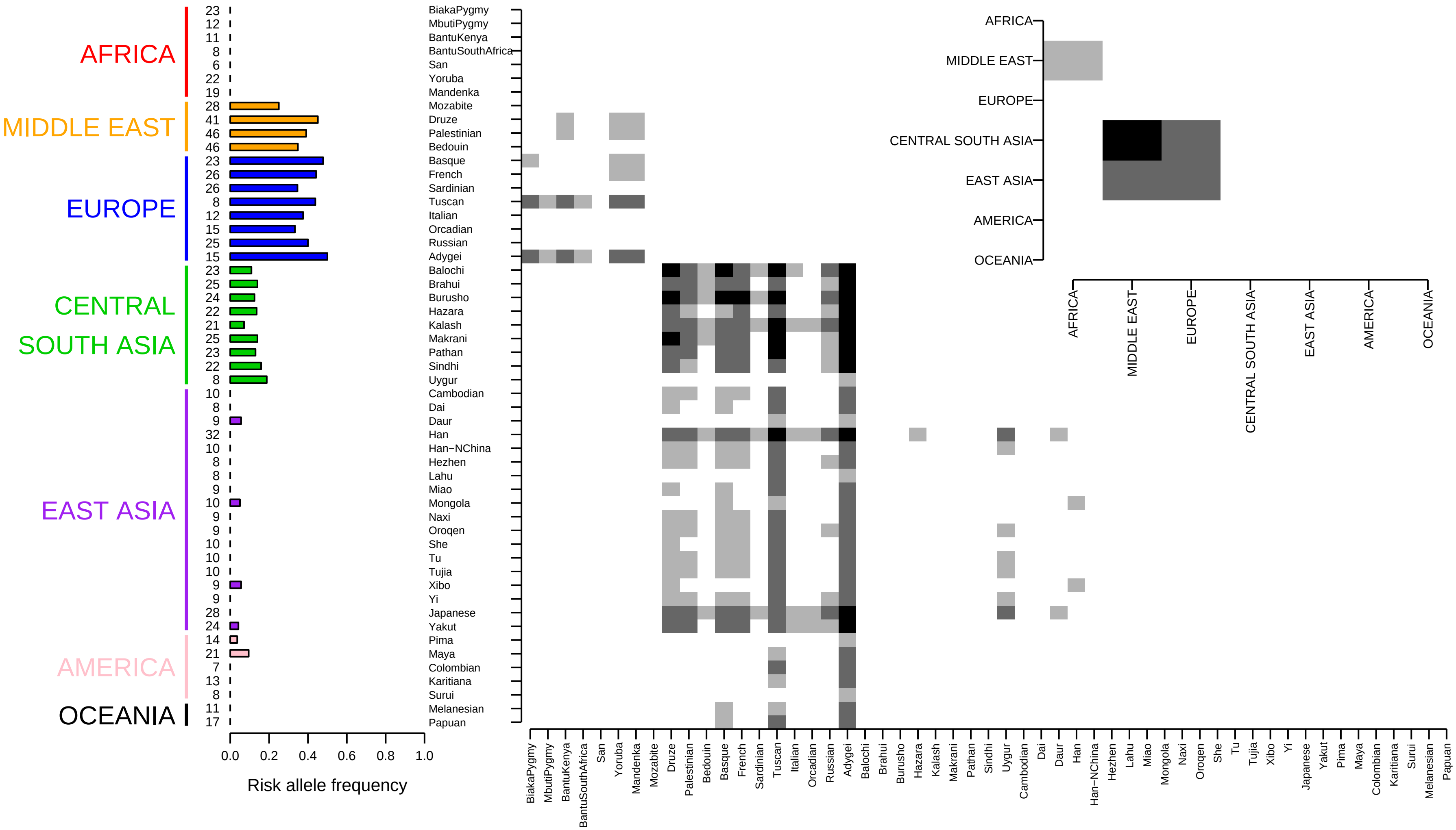

rs12708716 T1D

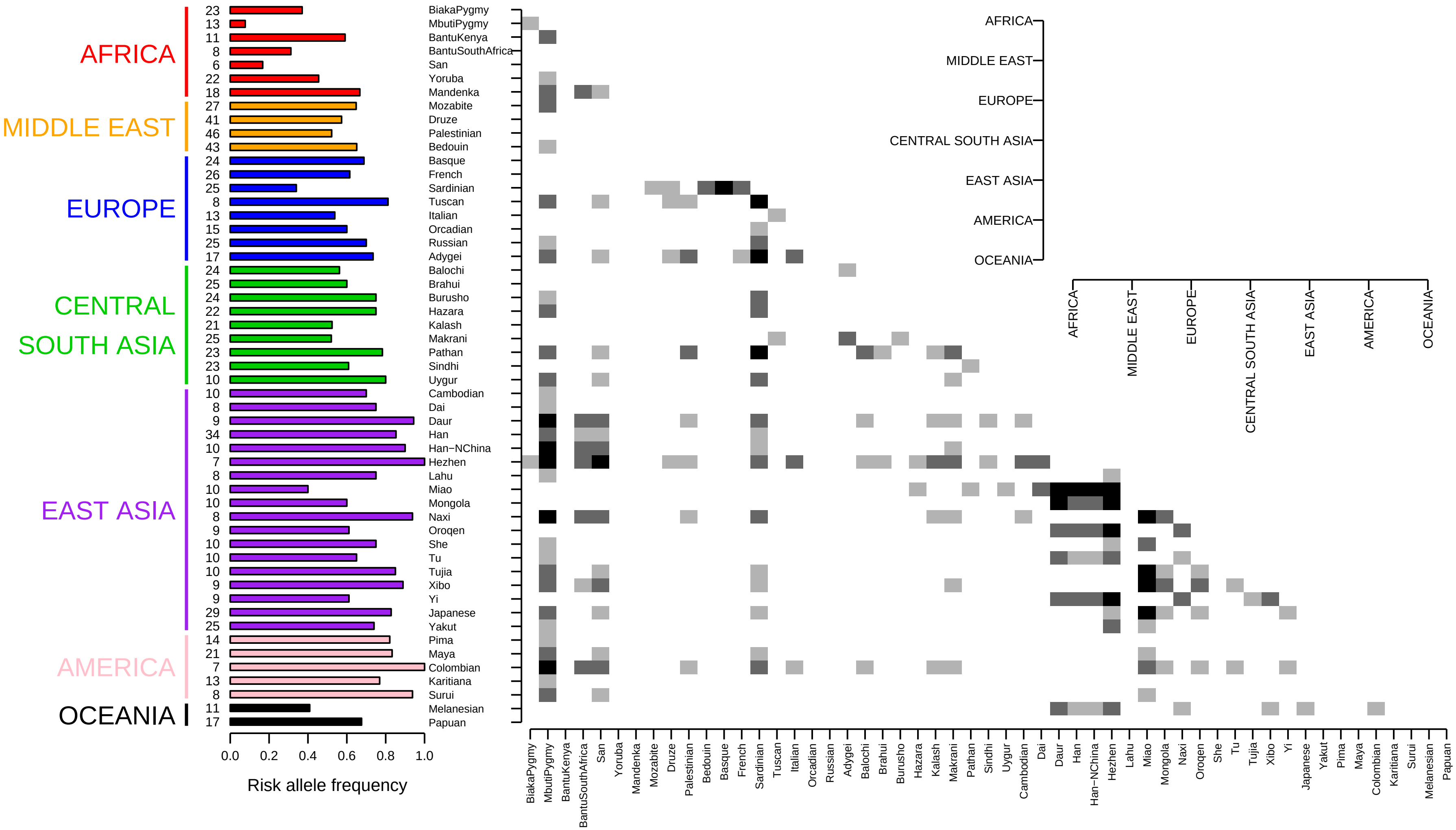

rs11171739 T1D

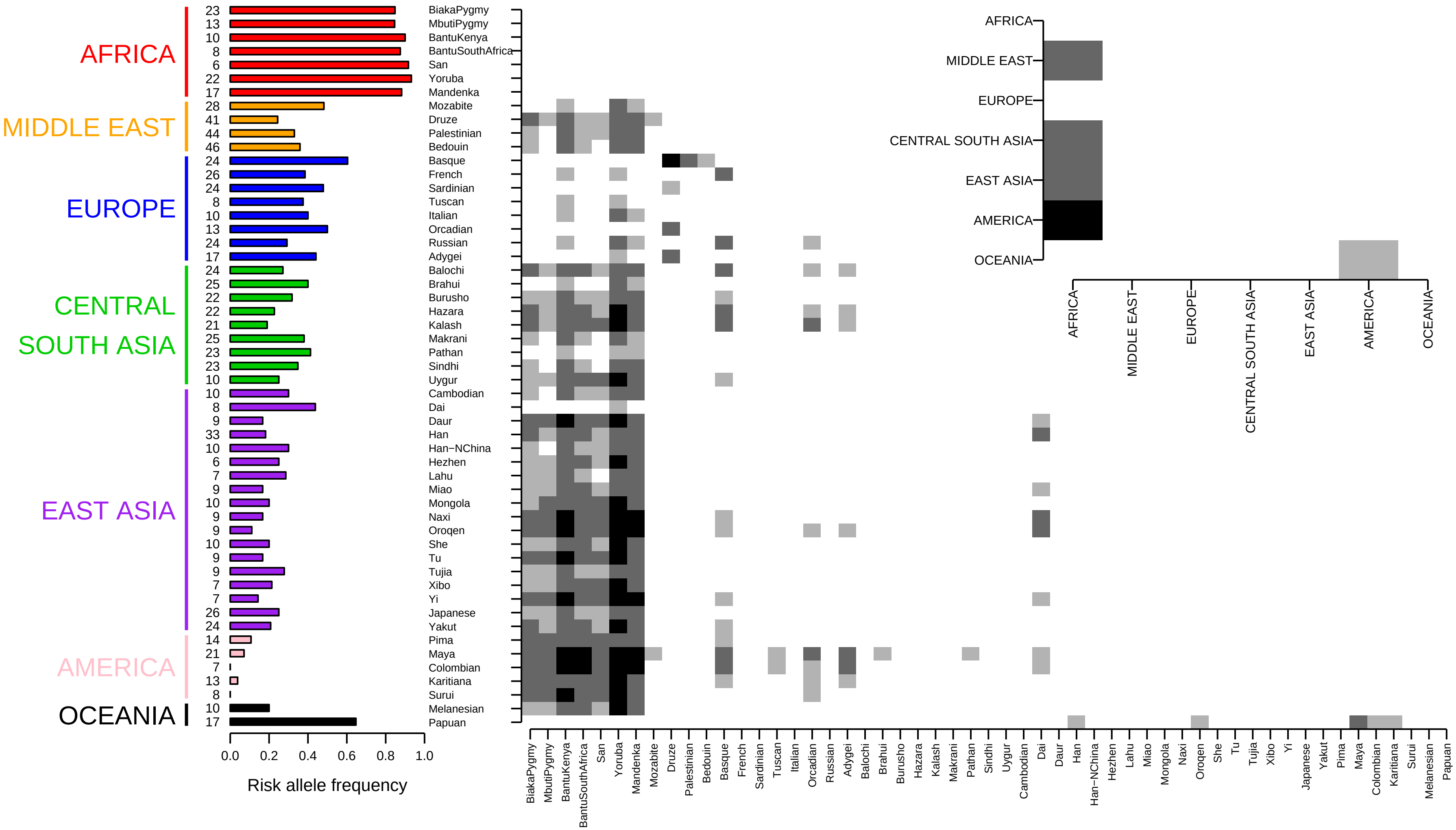

rs10946398 T2D

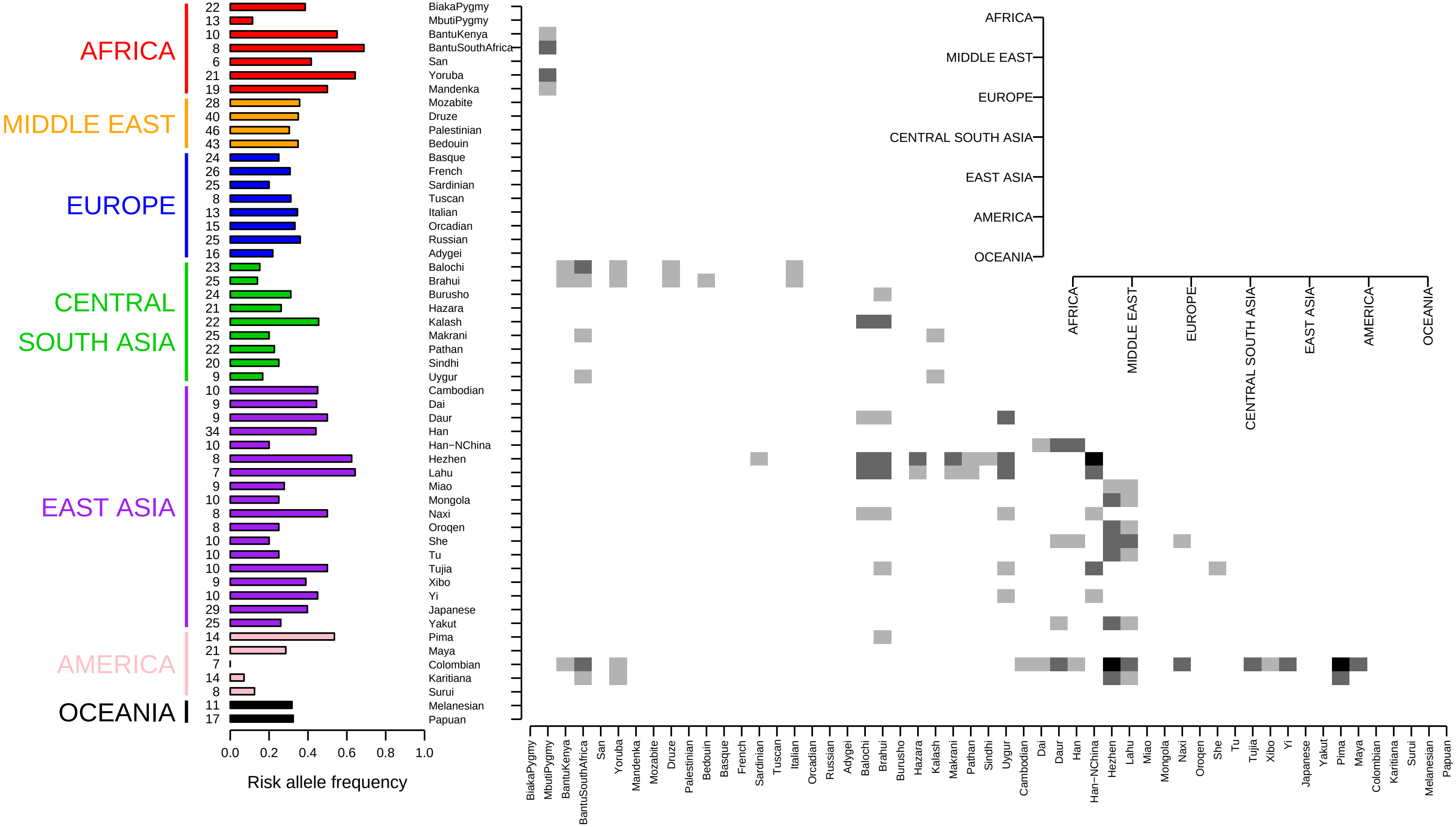

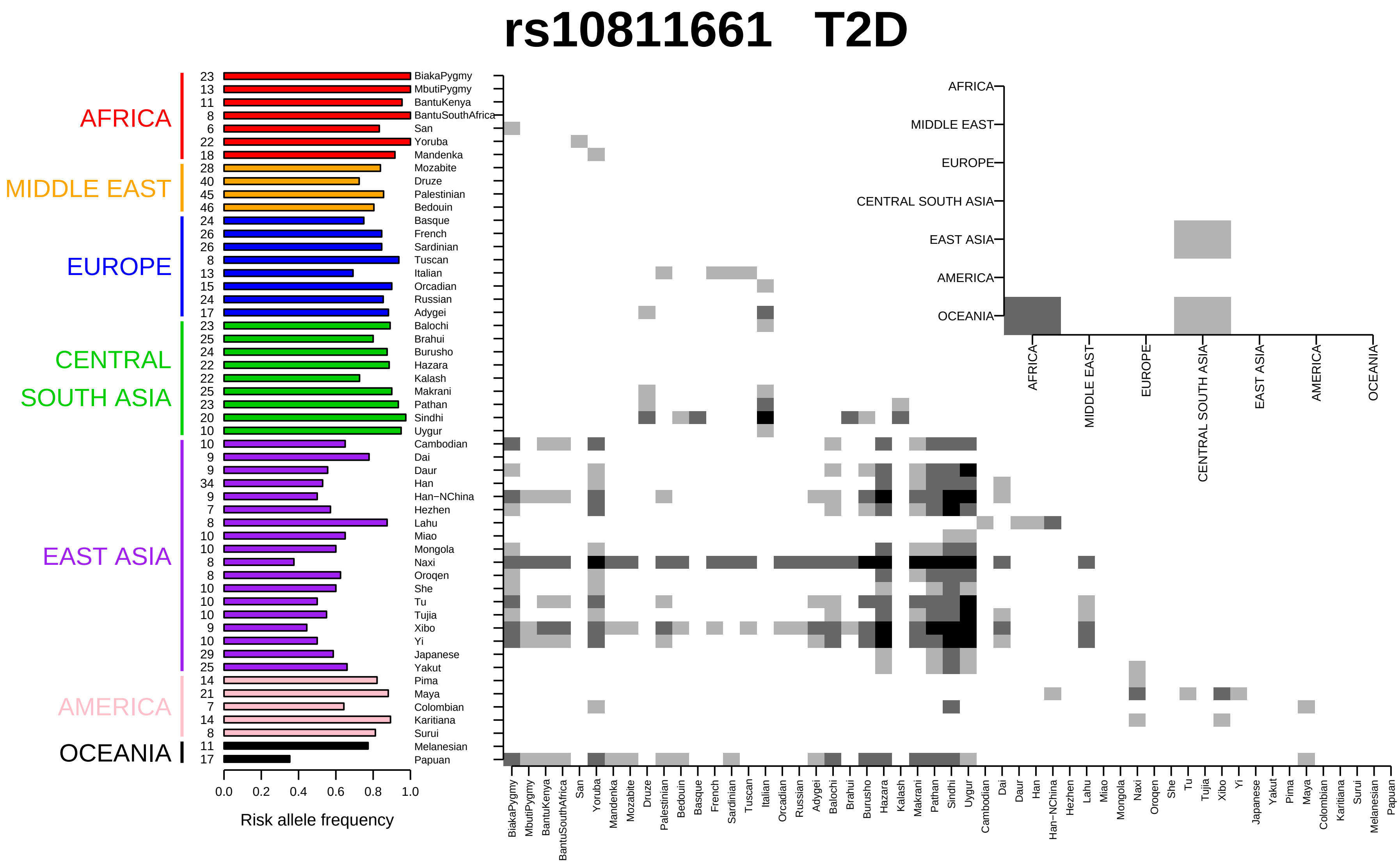
rs10811661 T2D
AFRICA
MIDDLE EAST
EUROPE
CENTRAL SOUTH ASIA
EAST ASIA
AMERICA
OCEANIA
23 BiakaPygmy
13 MbutiPygmy
11 BantuKenya
8 BantuSouthAfrica
6 San
22 Yoruba
18 Mandenka
28 Mozabite
40 Druze
45 Palestinian
46 Bedouin
24 Basque
26 French
26 Sardinian
8 Tuscan
13 Italian
15 Orcadian
24 Russian
17 Adygei
23 Balochi
25 Brahui
24 Burusho
22 Hazara
22 Kalash
25 Makrani
23 Pathan
20 Sindhi
10 Uygur
10 Cambodian
9 Dai
9 Daur
34 Han
9 Han−NChina
7 Hezhen
8 Lahu
10 Miao
10 Mongola
8 Naxi
8 Oroqen
10 She
10 Tu
10 Tujia
9 Xibo
10 Yi
29 Japanese
25 Yakut
14 Pima
21 Maya
7 Colombian
14 Karitiana
8 Surui
11 Melanesian
17 Papuan
0.0 0.2 0.4 0.6 0.8 1.0
Risk allele frequency

rs5215 T2D

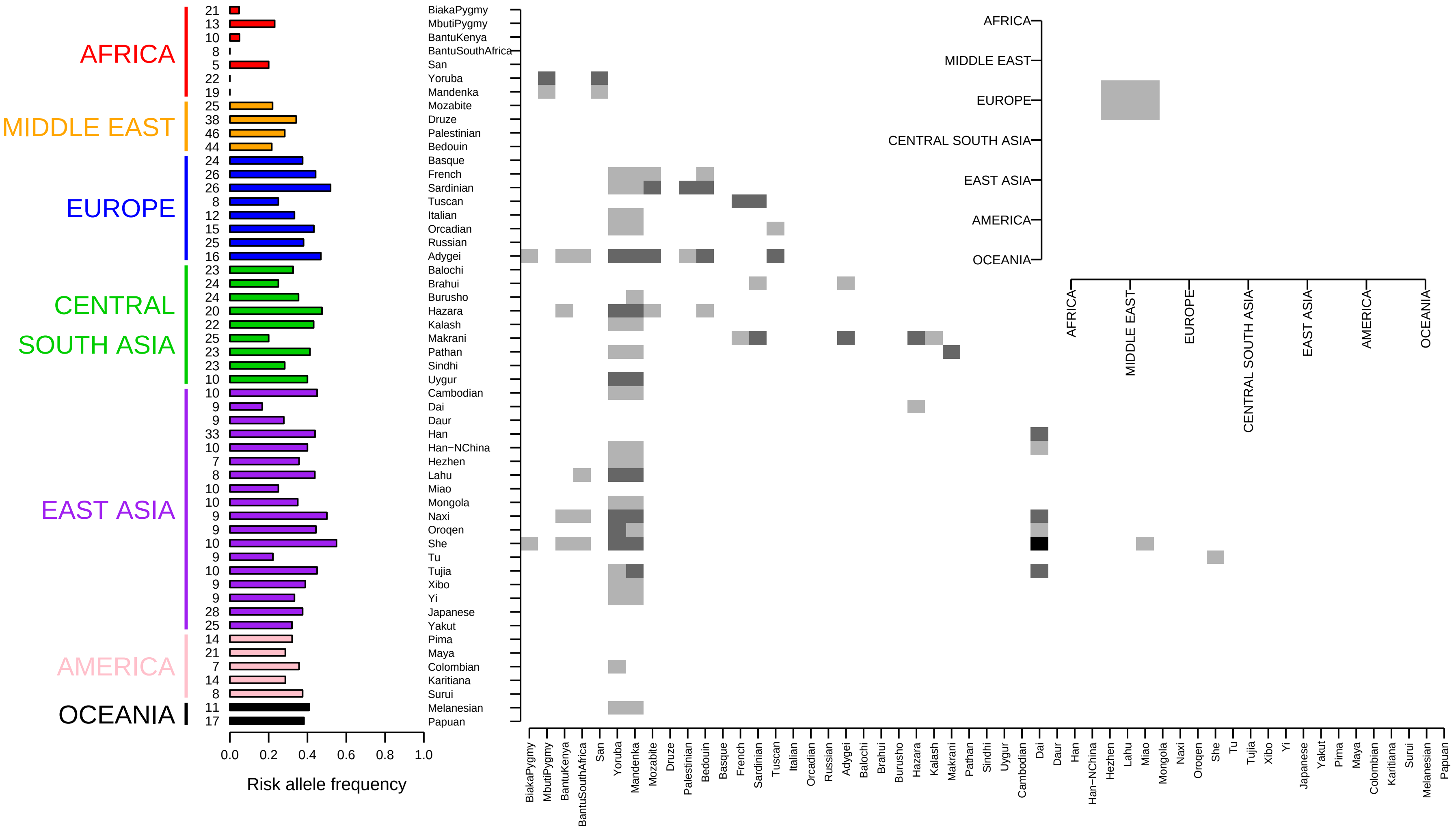
AFRICA
MIDDLE EAST
EUROPE
CENTRAL SOUTH ASIA
EAST ASIA
AMERICA
OCEANIA
21
13
10
8
5
22
19
25
38
46
44
24
26
26
8
12
15
25
16
23
24
24
20
22
25
23
23
10
10
9
9
33
10
7
8
10
10
9
9
10
9
10
9
9
28
25
14
21
7
14
8
11
17
0.0
0.2
0.4
0.6
0.8
1.0
Risk allele frequency
BiakaPygmy
MbutiPygmy
BantuKenya
BantuSouthAfrica
San
Yoruba
Mandenka
Mozabite
Druze
Palestinian
Bedouin
Basque
French
Sardinian
Tuscan
Italian
Orcadian
Russian
Adygei
Balochi
Brahui
Burusho
Hazara
Kalash
Makrani
Pathan
Sindhi
Uygur
Cambodian
Dai
Daur
Han
Han−NChina
Hezhen
Lahu
Miao
Mongola
Naxi
Oroqen
She
Tu
Tujia
Xibo
Yi
Japanese
Yakut
Pima
Maya
Colombian
Karitiana
Surui
Melanesian
Papuan

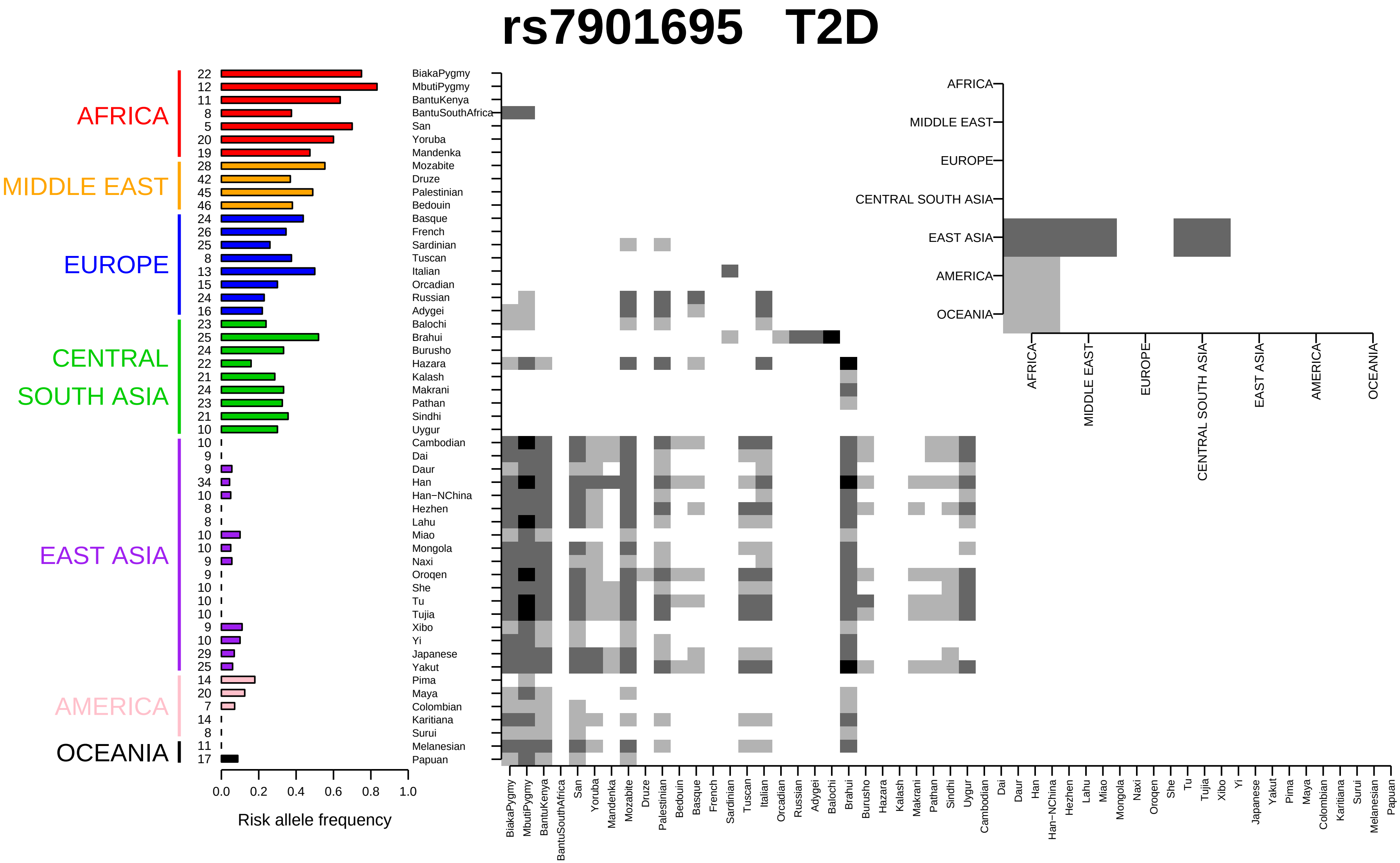
rs7901695 T2D
AFRICA
MIDDLE EAST
EUROPE
CENTRAL SOUTH ASIA
EAST ASIA
AMERICA
OCEANIA
Risk allele frequency
0.0 0.2 0.4 0.6 0.8 1.0
BiakaPygmy
MbutiPygmy
BantuKenya
BantuSouthAfrica
San
Yoruba
Mandenka
Mozabite
Druze
Palestinian
Bedouin
Basque
French
Sardinian
Tuscan
Italian
Orcadian
Russian
Adygei
Balochi
Brahui
Burusho
Hazara
Kalash
Makrani
Pathan
Sindhi
Uygur
Cambodian
Dai
Daur
Han
Han−NChina
Hezhen
Lahu
Miao
Mongola
Naxi
Oroqen
She
Tu
Tujia
Xibo
Yi
Japanese
Yakut
Pima
Maya
Colombian
Karitiana
Surui
Melanesian
Papuan

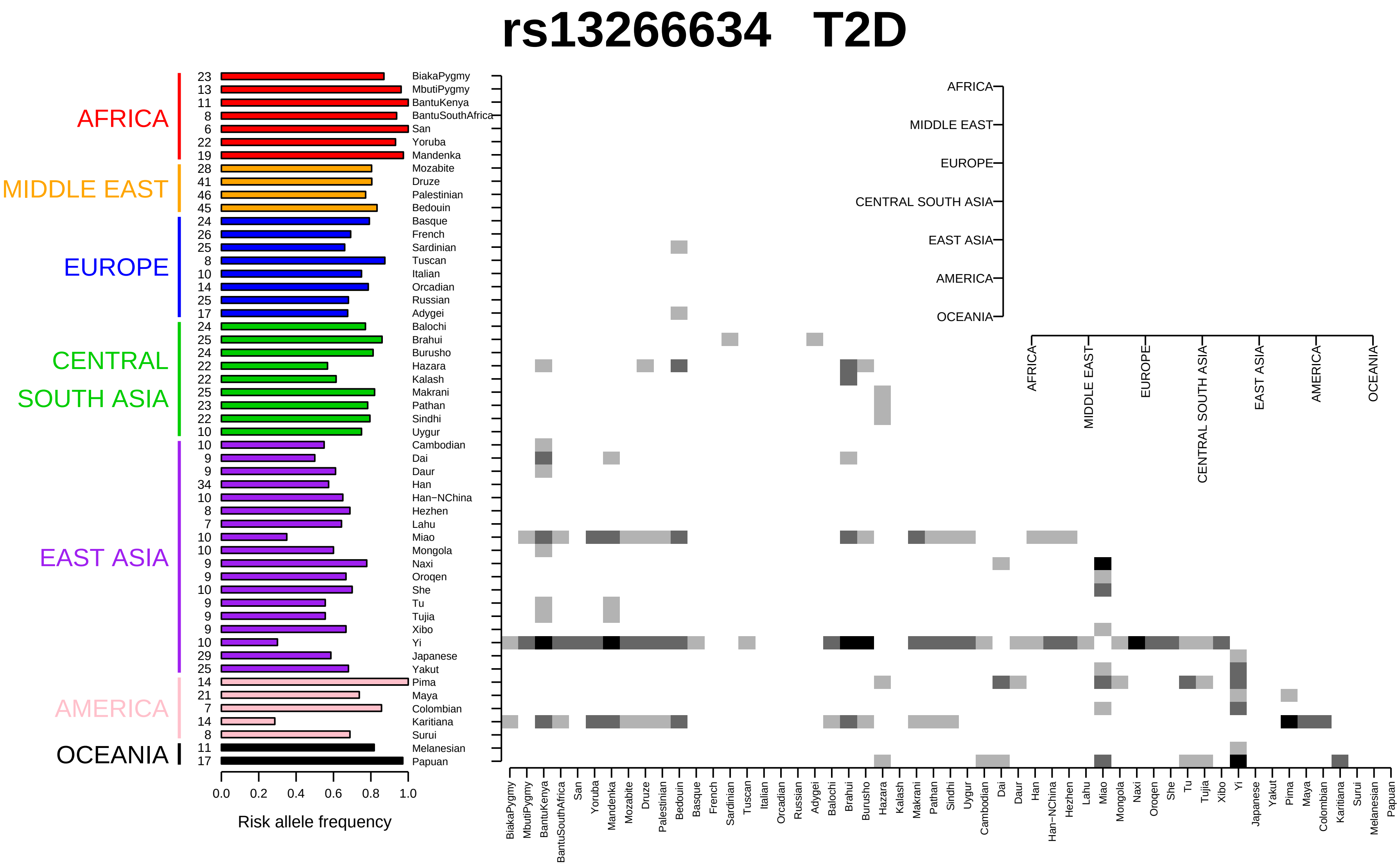
rs13266634 T2D
AFRICA
MIDDLE EAST
EUROPE
CENTRAL SOUTH ASIA
EAST ASIA
AMERICA
OCEANIA
23 BiakaPygmy
13 MbutiPygmy
11 BantuKenya
8 BantuSouthAfrica
6 San
22 Yoruba
19 Mandenka
28 Mozabite
41 Druze
46 Palestinian
45 Bedouin
24 Basque
26 French
25 Sardinian
8 Tuscan
10 Italian
14 Orcadian
25 Russian
17 Adygei
24 Balochi
25 Brahui
24 Burusho
22 Hazara
22 Kalash
25 Makrani
23 Pathan
22 Sindhi
10 Uygur
10 Cambodian
9 Dai
9 Daur
34 Han
10 Han−NChina
8 Hezhen
7 Lahu
10 Miao
10 Mongola
9 Naxi
9 Oroqen
10 She
9 Tu
9 Tujia
9 Xibo
10 Yi
29 Japanese
25 Yakut
14 Pima
21 Maya
7 Colombian
14 Karitiana
8 Surui
11 Melanesian
17 Papuan
0.0 0.2 0.4 0.6 0.8 1.0
Risk allele frequency
